# Endophytic Nanotechnology: An Approach to Study Scope and Potential Applications

**DOI:** 10.3389/fchem.2021.613343

**Published:** 2021-05-25

**Authors:** Mukesh Meena, Andleeb Zehra, Prashant Swapnil, Harish, Avinash Marwal, Garima Yadav, Priyankaraj Sonigra

**Affiliations:** ^1^ Laboratory of Phytopathology and Microbial Biotechnology, Department of Botany, Mohanlal Sukhadia University, Udaipur, India; ^2^ Centre of Advanced Study in Botany, Institute of Science, Banaras Hindu University, Varanasi, India; ^3^ Department of Botany, Acharya Narendra Dev College, University of Delhi, New Delhi, India; ^4^ Plant Biotechnology Laboratory, Department of Botany, Mohanlal Sukhadia University, Udaipur, India; ^5^ Department of Biotechnology, Vigyan Bhawan, Mohanlal Sukhadia University, Udaipur, India

**Keywords:** nanotechnology, nanoparticles, crop yield, genetic engineering, molecular approaches, gene carriers

## Abstract

Nanotechnology has become a very advanced and popular form of technology with huge potentials. Nanotechnology has been very well explored in the fields of electronics, automobiles, construction, medicine, and cosmetics, but the exploration of nanotecnology’s use in agriculture is still limited. Due to climate change, each year around 40% of crops face abiotic and biotic stress; with the global demand for food increasing, nanotechnology is seen as the best method to mitigate challenges in disease management in crops by reducing the use of chemical inputs such as herbicides, pesticides, and fungicides. The use of these toxic chemicals is potentially harmful to humans and the environment. Therefore, using NPs as fungicides/ bactericides or as nanofertilizers, due to their small size and high surface area with high reactivity, reduces the problems in plant disease management. There are several methods that have been used to synthesize NPs, such as physical and chemical methods. Specially, we need ecofriendly and nontoxic methods for the synthesis of NPs. Some biological organisms like plants, algae, yeast, bacteria, actinomycetes, and fungi have emerged as superlative candidates for the biological synthesis of NPs (also considered as green synthesis). Among these biological methods, endophytic microorganisms have been widely used to synthesize NPs with low metallic ions, which opens a new possibility on the edge of biological nanotechnology. In this review, we will have discussed the different methods of synthesis of NPs, such as top-down, bottom-up, and green synthesis (specially including endophytic microorganisms) methods, their mechanisms, different forms of NPs, such as magnesium oxide nanoparticles (MgO-NPs), copper nanoparticles (Cu-NPs), chitosan nanoparticles (CS-NPs), β-d-glucan nanoparticles (GNPs), and engineered nanoparticles (quantum dots, metalloids, nonmetals, carbon nanomaterials, dendrimers, and liposomes), and their molecular approaches in various aspects. At the molecular level, nanoparticles, such as mesoporous silica nanoparticles (MSN) and RNA-interference molecules, can also be used as molecular tools to carry genetic material during genetic engineering of plants. In plant disease management, NPs can be used as biosensors to diagnose the disease.

## Introduction

In recent years, nanomaterials have emerged as a novel type of material (Tayo, 2017; Hu et al., 2020). Nanotechnology is the latest technology with options for utilization in different fields like biology, sensing, medicine, chemistry and physics (Ramalingam et al., 2014; Ramalingam, 2019). Due to having various shapes and structures such as nanorods, nanospheres, nanocubes, nanobipyramids, nanobranches, nanoflowers, nanowires, nanocages, and nanoshells, nanomaterials appeared as the most stable materials (Li et al., 2015; Ramalingam et al., 2019; Xiao et al., 2019; Barupal et al., 2020a; Barupal et al., 2020b). Nanomaterials have unique electrical and optical properties that can be synthesized by different ways at low cost and have wide applications in several interdisciplinary branches of science (Gurav et al., 2019; Khan I. et al., 2019). Nano (dwarf) is the greek prefix which refers to the very small which in terms of nanoparticles, can refer to sizes up to 10^–9^ m i.e., one thousand millionth of a meter (Bayda et al., 2020; Chandran et al., 2020a; Chandran et al., 2020b). Nanotechnology belongs to the nanoscience in which nano-size molecules (1–100 nm) are utilized through practical applications using devices (Kumar and Kumbhat, 2016; Bayda et al., 2020). The term “nanotechnology” was first given by Taniguchi in 1974 to describe that which deals with the synthesis and application of nano-size particles (100 nm) (Filipponi, et al., 2010; Khan and Rizivi, 2014; El-Sayed and Kamel, 2020). According to the National Nanotechnology Initiative (NNI) United States, Nanotechnology is defined as a field of science, engineering, and technology where materials are practicised at the nanoscale size (1–100 nm), using unique phenomena in a wide range of biology, physics, chemistry, medicine, electronics and engineering fields (Chen et al., 2007; Lu et al., 2012; Kumari et al., 2018a; Kumari et al., 2018b). The most important properties of these nanoparticles (NPs) are their size which can manipulate the physiochemical and optical properties of a particular substance (Meena et al., 2015; Khan M. R. et al., 2019).

**FIGURE 1:**
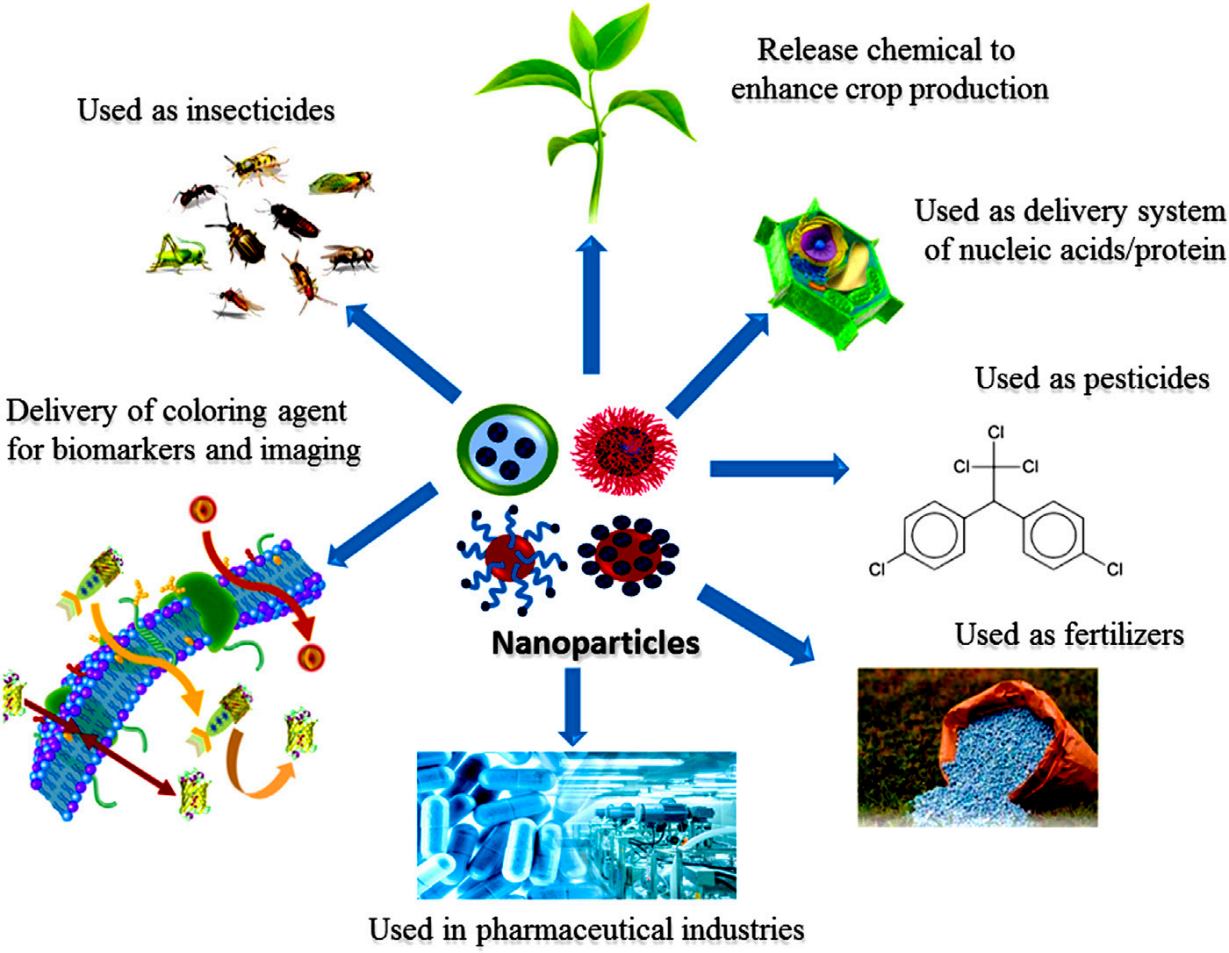
Various roles of nanoparticles.

Different NPs, such as gold (Au), silver (Ag), nickel (Ni), platinum (Pt), titanium (Ti), zinc (Zn), and palladiumn (Pd) are synthesized in various shapes and colors for the delivery of chemical, biological sensing, bioimaging (Dreaden et al., 2012; Bareket et al., 2016; Islam et al., 2018; Yew et al., 2020; Figure 1), gas sensing (Mansha et al., 2016; Ullah et al., 2017; Zhang H. et al., 2019), capturing of CO_2_ (Ramacharyulu et al., 2015; Ganesh et al., 2017), and other related applications. NPs are composed of three layers. The first layer known as surface layer which is composed of various types of small molecules, surfactants, metal ions, and polymers which functionalized the NPs. The second layer consists of as a shell layer, composed of different chemical materials as compared to the core. The core is the central part of the NP and generally refers to the NP itself (Shin et al., 2016; Heinz et al., 2017). Due to such remarkable characteristics, these materials gained considerable interest from researchers in multi-disciplinary areas. Mesoporousity imparts additional characteristics to NPs (Khan I. et al., 2019). In this review article, we provided a common overview related to NPs such as their different types, methods for synthesis, characterizations, properties, and their applications. The green synthesis of nanoparticles, specifically endophytic microorganism associated synthesis is a more beneficial method as compared to other physical and chemical methods such as top-down and bottom up methods, due to it being ecofriendly and cost-effective with significant morphology and size (Messaoudi and Bendahou, 2020). Micoorganism mediated synthesis of NPs is a challenging green process to manufacture NPs (Grasso et al., 2020). NPs are produced by microorganisms either through intracellular or extracellular process based on the location of enzymatic activity involved (Messaoudi and Bendahou, 2020). Microbial-mediated NPs synthesis showed advantage over the biosynthesis of NPs by algae and plants (Rana et al., 2020). Endophytic methods earned more attention in the field of medical, pharmaceuticals, environmental and agronomical applications (Gour and Jain, 2019; Rana et al., 2020). The last section of this review is used to discuss the future aspects and recommendations of NPs.

### Classification of Nanoparticles

Recently, various categories of NPs and their derivatives have been reported to have effective antimicrobial properties on the basis of their size, morphology and chemical properties. These derivatives of NPs can be Au, Ag, Cu, Ni, Pt, Ti, and Zn. Well-known classes of NPs have been described below on the basis of their physical and chemical characteristics.

### Organic Nanoparticles

Organic NPs are solid particles ranging between 10 nm to 1 μm in diameter and consist of organic compounds like polymeric or lipids (Kumar and Lal, 2014). Organic NPs received little attention as compared to inorganic NPs. In recent years, the pharmaceutical industries led the research into the synthesis of organic NPs. The search for nano-medicine developed well-established techniques to synthesize novel materials. They have an affinity for encapsulating or carrying active molecules as conjugates of proteins, vehicles for DNA delivery, liposomes, and co-polymer micelles (Ulbrich et al., 2016). Organic compounds are inherently and ultimately slow soluble in water or aqueous environments as compared to their inorganic counterparts, but organic NPs will not remain in the environment for a long period which makes them environmentally friendly (Yu et al., 2018). There are some well-known organic NPs that have been reported, such as dendrimers, micelles, liposomes, and ferritin, that showed some characteristics properties such as non-toxicity and biodegradability (Lee et al., 2012). Micelles and liposomes have a hollow core, also known as nanocapsules, and are considered as more sensitive to thermal and electromagnetic radiation such as heat and light (Ealias and Saravanakumar, 2017). Therefore, these unique characteristics make them an ideal alternative for drug delivery. The drug-carrying capacity, stability and delivery systems of organic NPs, either in the form of entrapped drugs or adsorbed drugs system determines their field applications and their effectiveness (Ealias and Saravanakumar, 2017; Meena et al., 2020a; Meena et al., 2020b). Organic NPs are also most widely used in the biomedical field (Yang et al., 2019).

### Carbon Nanomaterials

Carbon nanomaterials vary in shape, size, and function. There are three categories of carbon nanomaterials that are recognized: carbon nanotubes, graphene oxides, and fullerenes. The wall of carbon nanotubes can be single or multi, whereas graphene oxides and fullerenes are oxidized/reduced and C60 (buckyballs), respectively. Carbon nanomaterials are used in textiles engineering and medicines fields due to having antimicrobial activities against bacteria (Liu et al., 2009; Wang et al., 2013) and fungi (Sarlak et al., 2014; Wang X. et al., 2014), and also have been demonstrated and investigated as plant growth enhancers (Khodakovskaya et al., 2009; Tripathi et al., 2011; Wang et al., 2012; Elmer and White, 2018). Recently, it has been illustrated that carbon nanomaterials play a significant role in plant pathology. The reduced form of graphene oxide decreases 50% radial growth of *Aspergillus oryzae, Aspergillus niger,* and *Fusarium oxysporum* on agar plate at different concentration (100, 50, and 100 μg/ml) of graphene oxide. Single-walled carbon nanotubes were found to be more toxic to conidia of *Fusarium poae* and *Fusarium graminearum* (Wang X. et al., 2014). Nowadays, carbon nanotubes are explored as a phytosanitary treatment of pecan infected with *Xylella fastidiosa* (Hilton et al., 2017). Carbon nanomaterials open new areas of microbiological research by uncovering microbial growth inhibition mechanisms (Liu et al., 2009; Sawangphruk et al., 2012; Chen et al., 2013; Berry et al., 2014; Wang Y. et al., 2014). In *Fusarium* sp. the inhibition mechanism is governed by carbon nanotubes (single-walled) through the mechanism of water uptake and plasmolysis induction (Wang L. et al., 2017). Certain forms of carbon nanomaterials can be produced for antimicrobial activity at relatively low cost and it attracts researchers to develop an evaluation study for carbon nanomaterials in agriculture and other fields (Zehra et al., 2015).

### Inorganic Nanoparticles

NPs composed of metal and metal oxide are generally classified as inorganic NPs, which are discussed below.

### Metal-Based Nanoparticles

The NPs have characteristic properties such as sizes as low as 10–100 nm, pore size, high surface area to volume ratio, surface charge and density, amorphous and crystalline structures, spherical or cylindrical in shape, colored, high reactivity, and their sensitivity to environmental factors like moisture, air, heat, and sunlight (Ealias and Saravanakumar, 2017). Metal-based NPs are synthesized from metals such as aluminium (Al), cadmium (Cd), cobalt (Co), copper (Cu), gold (Au), iron (Fe), lead (Pb), silver (Ag) and zinc (Zn) (Monych et al., 2019) and can exist in solutions. These NPs have gained much attention in pharmaceutical industries for their use in manufacturing medicines (Padrela et al., 2018). These NPs can be modified by altering their chemical groups to binds with antibodies (Ruiz et al., 2019). Some noble metals such as Ag-, Au-, and Pt- synthesized NPs have specific properties which were used in biomedical fields to cure diseases (Kim et al., 2018). Therefore, these NPs used to prepare drugs had anticancer, radiotherapy enhancement, drug delivery, thermal ablation, antibacterial, diagnostic assays, antifungal, gene delivery, and many other properties (Jahangirian et al., 2017; Sharma et al., 2018). Fan et al. (2018) reported that metal NPs can be target to different cells along with different functional groups, such as peptides, antibodies, RNA, and DNA, and with potential biocompatible polymers (polyethylene glycol; PEG). L-Ascorbic acid has been used to synthesize Cu-NPs with size >2 nm as an antibacterial agent against Gram-negative and Gram-positive bacteria and has been reported as a stabilizer and reducing agent (Tomar and Garg, 2013; Meena et al., 2018; Sathiyabama and Manikandan, 2018).

Au NPs were found useful in the identification of different microorganisms by detecting and evaluating DNA and identifying protein interactions from biological samples. Au-NPs have been used widely and help to detect cancerous cells through bioimaging (Dreaden et al., 2012). They can be synthesized by different processes but are currently being produced by *Pseudomonas* endophytic microorganisms such as *Pseudomonas fluorescens* 417 or *Fusarium solani* (Syed et al., 2016; Clarance et al., 2020). Ag-NPs combined with amoxicillin, penicillin G, clindamycin, vancomycin, and erythromycin showed antimicrobial activities against the pathogenic strains (Rai et al., 2012). Ag NPs play a very important role in biomedicine, performing cell imaging, cancer therapy, genetic delivery, drug delivery, and different disease diagnose (Keat et al., 2015; Shanmuganathan et al., 2019). There are many endpohytic microorganisms that have been involved in AgNPs synthesis such as *Bacillus siamensis* C1, *Pseudomonas poae* CO, *Aneurinibacillus migulanus*, and *Alternaria* sp. (Ibrahim et al., 2020). Silicon (Si) nano substrates with Ag- or Cu-NPs showed antibacterial activities against *Escherichia coli* (Fellahi et al., 2013; Shahriary et al., 2018). It has been found that Si coated by Ag is highly biocompatible in the human lung, especially adenocarcinoma epithelial cells, whereas the Cu-coated Si showed high cytotoxicity which may lead to death (Wu et al., 2017). Pd-NPs are more prolific and act as anticancer and stabilizing agents and are used by many pharmaceutical industries to produce medicines (Siddiqi and Husen, 2017; Yaqoob et al., 2020). NPs are being used by many pharmaceutical industries and gained more attention in research fields (Gao and Lowry, 2018).

### Metal Oxide–Based Nanoparticles

The metal oxide based NPs such as Ag_2_O, FeO, MnO_2_, CuO, Bi_2_O_3_, ZnO, MgO, TiO_2_, CaO, and Al_2_O_3_, enhance their activity and were found to have potent antibacterial activities (Yaqoob et al., 2020). The oxide of Ag-NPs (Ag_2_O) was recommended as a novel source of antibiotics (Torabi et al., 2020) and showed antibiotic properties against *E. coli* (Salas-Orozco et al., 2019). Whereas, ZnO NPs also showed antibacterial activities against high pressure and temperature tolerant Gram-positive microorganisms (*Staphylococcus aureus* and *Bacillus subtilis*), Gram-negative microorganisms (*E. coli*, *Pseudomonas aeruginosa*) and spores of *Peronospora tabacina* as compared to CuO and Fe_2_O_3_, NPs respectively (Azam et al., 2012a, b; Prasanna et al., 2019; Wagner et al., 2016). The antibacterial activity of ZnO NPs is inversely proportional to their size (Prasanna et al., 2019). While in the case of TiO_2_-NPs, its antibacterial activity depends upon its morphology, crystal structure, size, and shape. TiO_2_ emerged as an important antibacterial agent by enhancing the anti-microorganism effect of tetracycline, β-glycopeptides, aminoglycosides, lactums, cephalosporins, and macrolids against methicillin-resistant *Staphylococcus aureus* (Roy et al., 2010). It has been also reported that TiO_2_-NPs enhanced the antifungal activity against *Candida albicans* biofilms (Haghighi et al., 2013).

CuO-NPs have significant antimicrobial properties against *Enterococcus faecalis* and *E. coli* as compared to other various bacterial strains like *Klebsiella pneumoniae*, *Proteus vulgaris*, *Shigella flexneri*, *Salmonella typhimurium*, *P. aeruginosa*, and *Staphylococcus aureus* (Ahamed et al., 2014). As ZnO, CuO, Ag_2_O, Fe_2_O_3_, and TiO_2_ and some other metal oxide based NPs such as MnO_2_, Bi_2_O_3_, and FeO also showed their beneficial activity in biomedical fields through their use in drug delivery, bioimaging, and antimicrobial activities. FeO-NPs (4.8 nm) showed their higher relativity value (444.56 mM^−1^ s^−1^) in the bioimaging of tumor cells (Wang L. et al., 2017; Gao et al., 2018). Some NPs (MnO_2_) are very significant for medical applications such as bioimaging, biosensing, cancer therapy, molecular adsorption, and drug delivery due to their physicochemical, structural, and morphological-based properties (Chen et al., 2019a; Wu et al., 2019). MnO_2_ has been considered as a novel compound due to having lower cytotoxicity and higher hemo/histocompatibility. Bi_2_O_3_-NPs (35 nm) is recommended for use with phenothiazine photosensitizer for cancer treatment and in drug delivery (Ovsyannikov et al., 2015; Szostak et al., 2019). Some NPs show their activity under a specific environment; for example, CaO and MgO, show their anti-bacterial activity under alkaline and oxygenic environments and are considered as excellent biocompatible NPs. MgO-NPs have been studied as antibacterial agents against *E. coli and Staphylococcus aureus* under oxygenic conditions (Leung et al., 2016; Meena et al., 2019a; Meena et al., 2019b). Metal oxide based NPs can be synthesized at low cost using simply accessible materials and, can also be utilized in food processing and environmental conservation with biomedical uses. These NPs have excellent properties when compared with their metal counterparts.

### Doped Metal/Metal/Metal Oxide–Based NPs

NPs can be modified chemically to make more stable materials that are safe for the ecosystem. The antimicrobial activities of ZnO-NPs against *B. subtilis*, *Staphylococcus aureus*, *E. coli*, and *P. aeruginosa* can be increased approximately by 5% by doping with Mg (magnesium), Sb (antimony) or Ta (tantalum) as compared to ZnO-NPs and have less self-toxicity issues (Guo et al., 2015). The considerable improvement by approximately 10,000 times was pragmatic in the antimicrobial activity of Zn and CuO-doped NPs as compared to the pure oxide of Cu- and Zn-NPs on the surface of cotton fabric by ultrasound irradiation (Malka et al., 2013). Doped Mn/ZnO NPs have been used to study the antibacterial and photocatalytic activity in pure ZnO-NPs by observing its optical properties and structural morphology; it was found that doped NPs showed more activity (Alshehri and Malik, 2019). TiO_2_ doped with Cu_2_O in the presence of rGO results in improved antimicrobial activity with a higher inhibition zone for microorganisms as compared to pure TiO_2_ (Wu, 2017). In biomedical applications, Ag-doped MgO emerged as a significant antimicrobial agent as compared to pure oxides of Mg against *Staphylococcus aureus* and *P. aeruginosa* (Llorens et al., 2012; Nganga et al., 2013; Meena and Swapnil, 2019). Ag- and carbon-monolith-doped NPs were also found to be more active antimicrobial agents against *C. albicans*, *Staphylococcus aureus*, and *E. coli* (Arakawa et al., 2019). Disk diffusion analyses have been performed against assured disease-causing pathogens like *Staphylococcus aureus*, *E. coli*, *B. cereus*, and *P. aeruginosa* to analyze their antimicrobial effects (Zhu et al., 2019). Therefore, doped metal-oxide based NPs showed more activity as antimicrobial agents as compared to pure oxides (Ewald et al., 2011).

### Metal Sulfide–Based Nanoparticles

To protect the surface of the NPs, the amalgamation of semiconductor metal sulfide NPs into polymers has been performed through chemical methods (Mthethwa et al., 2011). Poly methyl methacrylate (PMMA) has been considered one of the most extensively studied polymers among a vast variety of available polymers due to having significant chemicophysical and mechanical properties (Kumar et al., 2019; Gross et al., 2007). Therefore, researches are focused on the synthesis of metal sulfides/polymer nanocomposites (ZnS/PMMA and CdS/PMMA), their characterization, and their optical properties *via.* direct blending to attain optically clear and thermally stable compounds with good mechanical properties (Thanh and Green, 2010; Agrawal et al., 2011; Ezhov et al., 2011; Hashmi, 2012; Prabhu and Pattabi, 2012; Ajibade and Mbese, 2014). Metal-based chalcogenides such as PbS, CdSe, CdSe-CdTe, and CdSe-ZnTe have multifarious structures (Li and Wong, 2017; Meena et al., 2016a; Meena et al., 2016b). Metallic sulfides containing chalcogenide sulfur have been analyzed and have emerged as an important toxic-free metal, earing much attention in the biomedical field (Dahoumane et al., 2016). AgS, FeS, CuS, and ZnS have been studied as the most well-known metal sulfides for biomedical applications in photothermal therapy, biosensing, drug deliveries, and biomolecular imaging (Goel et al., 2014). CuS-NPs and their derivatives have been widely used in molecule detection technology as metabolites (glucose) detectors, DNA detectors, and food-based pathogen detectors. The metal-sulphide based NPs got recognition in the field of biosensing which promotes electron transfer reactions.

Furthermore, CuS was exposed to anthropological immunoglobulin A (IgA) as a thin film–based immunosensor (Attarde and Pandit, 2020) and photothermal agents for the treatment of cancerous cells (Tian et al., 2011). Ag_2_S quantum dots have been used in the tracking and designing of cells *in vivo*, bioimaging, photodynamic treatment, and diagnostic purposes. Ag_2_S quantum dots can also be used as a significant active tracker for human mesenchymal stem cells (MSCs) and are also considered as antimicrobial agents (Meena et al., 2016c; Argueta-Figueroa et al., 2017). According to Ding et al. (2016), Fe_3_S_4_ showed pseudoenzyme activities to enterprise a measurable photometric enzyme and assess in human serum, which is oxidized by hydrogen peroxide through Fe_3_S_4_ NPs.

### Synthesis of Nanoparticles

There are several methods that can be employed for the synthesis of NPs, which are most often divided into two main categories Bottom-up methods and Top-down methods (Wang and Xia, 2004; Meena et al., 2017a, b; Kishen et al., 2020).

**FIGURE 2:**
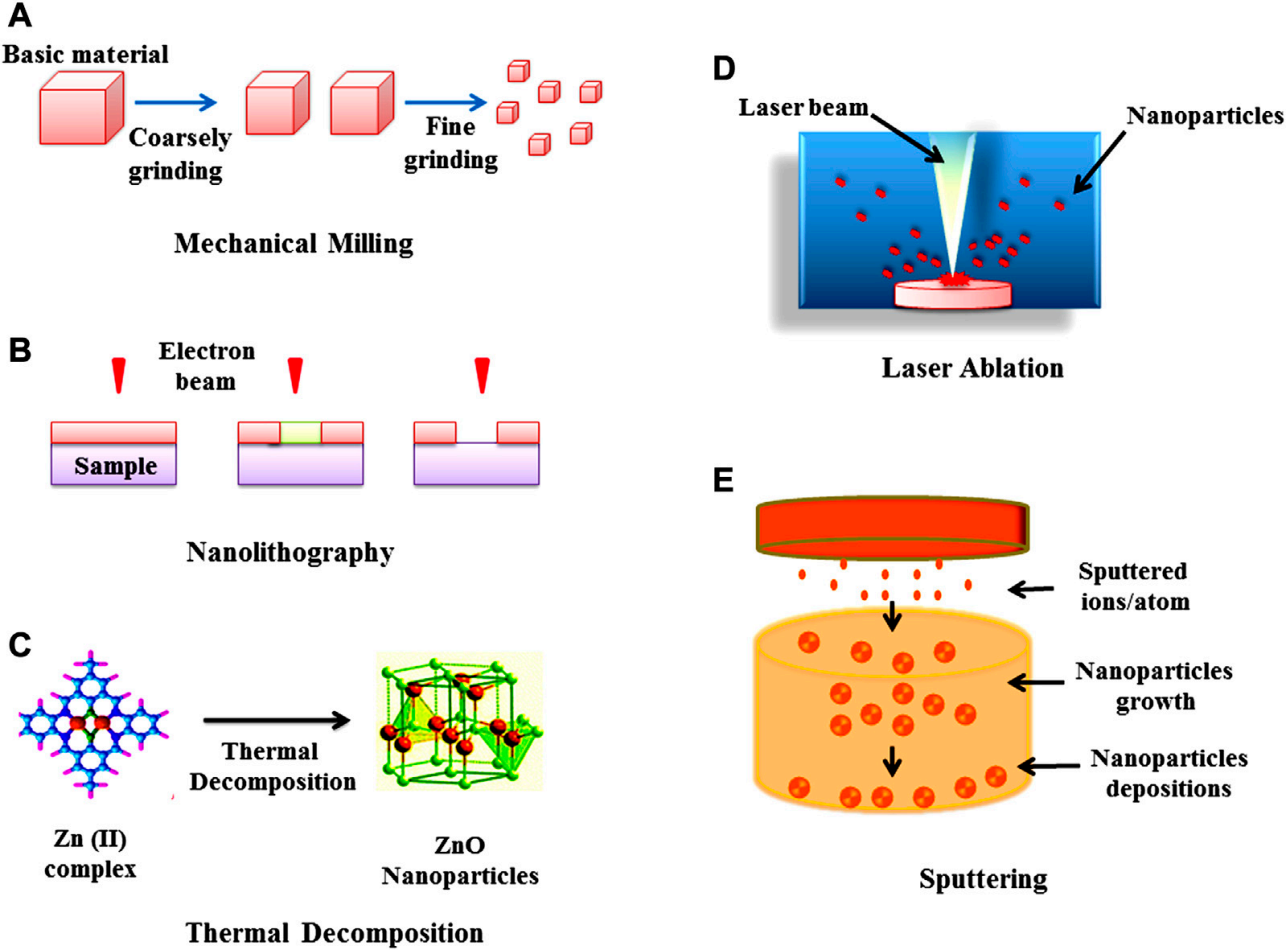
Top-down approach for the synthesis of nanoparticles **(A)** Mechanical milling **(B)** Nanolithography **(C)** Thermal decomposition **(D)** Laser ablation, and **(E)** Sputtering.

**FIGURE 3:**
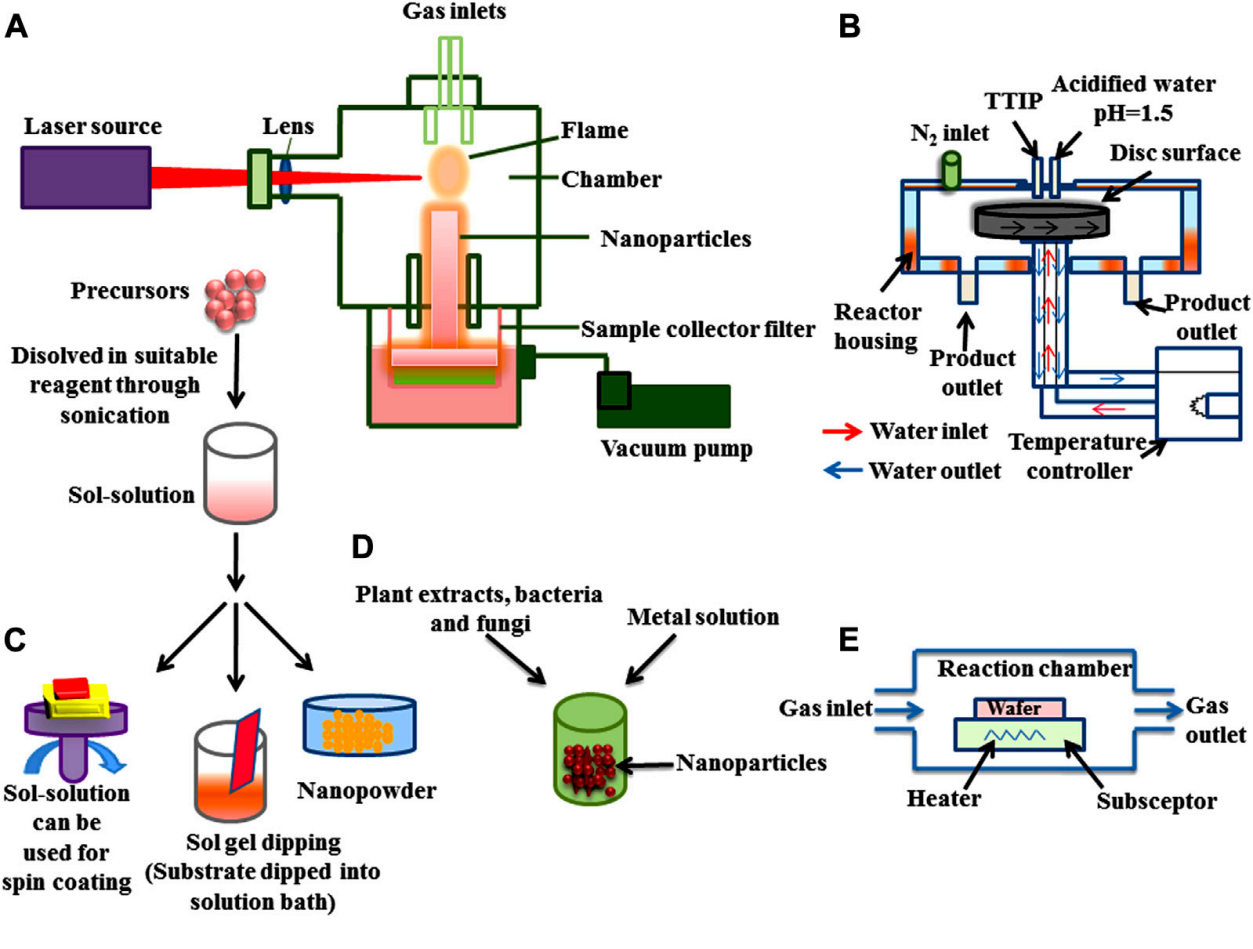
Bottom-up approach for the synthesis of nanoparticles **(A)** Pyrolysis **(B)** Spinning **(C)** Sol gel **(D)** Green synthesis, and **(E)** Chemical vapour deposition (CVD).

In the top-down method (or destructive method) NPs are synthesized by decomposition of larger units into smaller units and these smaller units are further converted into appropriate NPs (nanometric scale particles) (Conf, 2017). This method is followed by various types of processes such as mechanical milling (Liversidge and Cundy, 1995; Merisko-Liversidge et al., 2003; Yadav et al., 2012), nanolithography, laser ablation (Hulteen et al., 1999; Amendola and Meneghetti, 2009) sputtering and thermal decomposition (Chrissafis and Bikiaris, 2011; Verma et al., 2018; Araújo et al., 2018) which have been described in Figure 2. While in bottom-up synthesis (physicochemical processes) NPs such as polymersomes (Kapakoglou et al., 2008; Christian et al., 2009), micelles (Zhu et al., 2011), liposomes and vesicles (Camelo et al., 2009) polymer conjugates (Grover and Maynard, 2010), capsules (Delcea et al., 2010; Moraes et al., 2011; Zhao et al., 2011), polymeric NPs (Grabnar and Kristl, 2011) and dendrimers (Ravoo, 2008) are synthesized by several processes like sol-gel method, green synthesis, spinning, and chemical vapour deposition (CVD) pyrolysis (Mann et al., 1997; Yarema et al., 2010; Iravani, 2011; Biswas et al., 2012; Ramesh et al., 2013; Mogilevsky et al., 2014; Liu D. et al., 2015; Needham et al., 2016; Parveen and Tremiliosi-Filho, 2016). These methods have been illustrated in Figure 3.

Amongst the above-mentioned methods, the green synthesis method has emerged as the most beneficial method (Iravani, 2011; Patra and Baek, 2014; Kitching et al., 2015; Park et al., 2016; Singh et al., 2016; Dahoumane et al., 2017; Singh et al., 2017). Green synthesis utilizes different metals which have been applied in different fields, such as medical (Shah et al., 2015; Al-Sheddi et al., 2018). The biological metallic NPs are synthesized by *Nepeta deflersiana* (Al-Sheddi et al., 2018), pink yeast, and *Rhodotorula* sp. ATL72 (Soliman et al., 2018) to cure various disorders in medical fields, for their antimicrobial activity, as sensors for various biomolecules, for gene delivery, and for labeling of cells in medicine and plants (Wang et al., 2006; Khandel et al., 2018).

### Mechanisms of Microorganism Based Nanoparticle Biosynthesis

**FIGURE 4:**
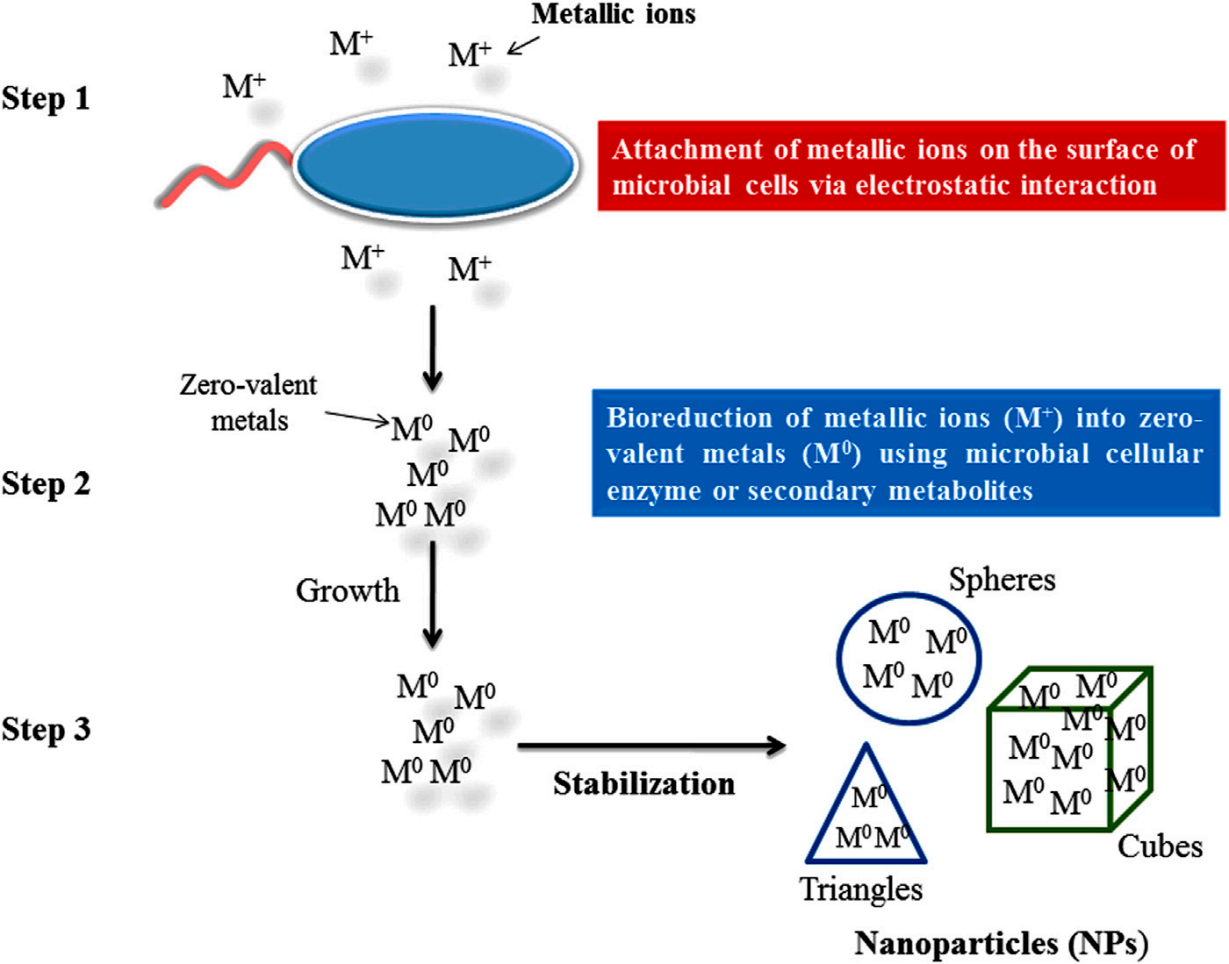
Steps in the green synthesis mechanism of nanoparticles.

**FIGURE 5:**
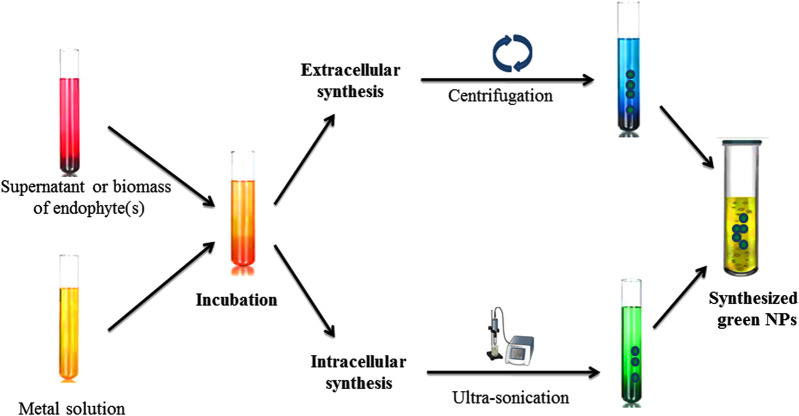
Synthesis of nanoparticles using endophytic microorganisms.

To reduce the metal ions into NPs, secondary metabolites secretion and intra and extra microbial enzyme (cellulary) play important roles. Under metal ion stress, microorganisms secrete enzymes and biomolecules which reduce the effect of metal ions and the toxicity of metal ions are then reduced by detoxification (Singh A. et al., 2018). There are three steps which have been reported for the biosynthesis of NPs by microorganisms shown in Figures 4, 5.

### Metal Ions and Microbial Interaction

Through electrostatic interaction metallic ions attach to the negatively charged surface of a microbial cell wall and are transported inside the cell through cationic membrane transport systems (Ghashghaei and Emtiazi, 2015; Singh A. et al., 2018).

### Bio-Reduction of Metallic Ions

Metallic ions can be bioreduced either by functional group (hydroxyl group or carboxyl group) associated with biomolecules having reduction capabilities or by microbial enzymes (NADH-dependent nitrate reductase) which catalyze the reduction of Ag ions to Ag NPs (Talekar et al., 2012; Velusamy et al., 2016). In this reaction, a mono or di valant oxidation state is converted into a zero valent oxidation state. After reduction, zero valent state AgNPs associate to form various morphological shaped (ovale, spheres, cubes, triangles, hexagons, etc.) NPs (Chokkareddy and Redhi, 2018).

### Stabilization of NPs

This step is followed to stabilize the shape of biosynthesized NPs by preventing further growth and agglomeration (Singh A. et al., 2018) by controlled and optimized physicochemical parameters such as metal salt concentration, temperature, incubation period, pH, agitation, or concentration and nature of nutrients (carbon and nitrogen) in culture media (Khandel et al., 2018). The small size and particular shape of NPs biosynthesized by endophytic microorganisms provide good quality and a higher surface/volume ration which affects the activity positively (Niño-Martínez et al., 2019).

### Nanoparticles Synthetized by Endophytic Microorganisms

**FIGURE 6:**
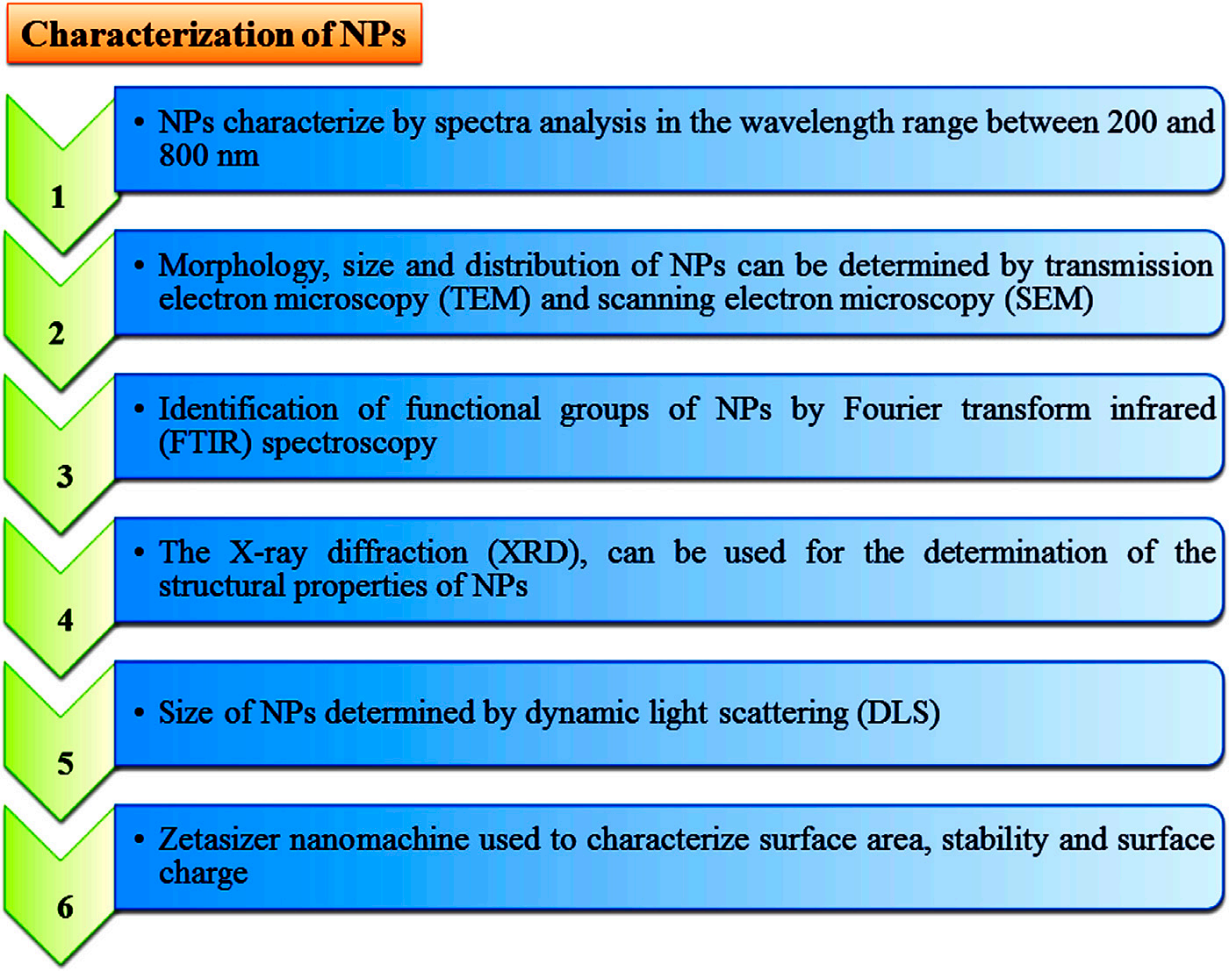
Different characterization techniques to analyse properties of nanoparticles.

Green synthesis or biological methods to synthesize metal NPs are becoming more popular. Among them, endophytic microorganisms such as bacteria, fungi, and actinomycetes have the tendency to convert metal ions into metallic NPs such as Ag, Au, Zn, and Cu with the help of secondary metabolites and cellular enzymes (Joshi et al., 2017; Soliman et al., 2018). Endophytic bacteria under high metallic ion stress establish the defense mechanism to reduce the toxicity of metal ions through the precipitation of metallic ions at the nanometer scale to synthesize NPs (Iravani et al., 2014). Due to having metallic ion stress tolerance tendency, endophytic bacteria emerged as good entrant for NPs synthesis (Syed et al., 2019). Ag NPs with antibiofilm, antibacterial and antifungal activity can be synthesized from *Bacillus siamensis* C1, *Pseudomonas poae* CO (Ibrahim et al., 2019; Ibrahim et al., 2020), or *Aneurinibacillus migulanus* (Prathna et al., 2010), while Au NPs (5–50 nm) synthesized by *Pseudomonas fluorescens* 417 have bactericidal activity (Syed et al., 2016). Ag NPs synthesized by *Pseudomonas aeruginosa* were reported as higher active NPs. Due to having metal uptake, their accumulation and toleration capable endophytic fungi attracted more attention in research fields (Moghaddam et al., 2015). There are several advantages to endophytic fungi which make it a better microorganism for NPs’ synthesis, such as trouble-free isolation from plants or soil (Xiaowen et al., 2019), more secretion of metabolites and extracellular enzymes for the reduction of metallic ions into NPs, and it being easy to grow rapidly. Au NPs synthesized through the isolation of *Fusarium solani* from *Chonemorpha fragrans* can be used to cure cervical cancer cells (Clarance et al., 2020). ZnO NPs (size ranges from 15 to 45 nm) are synthesized by culture filtrate of the *Alternaria tenuissima* (Abdelhakim et al., 2020). *Exserohilum rostrata* has been used to synthesize Ag NPs (size ranges from 15 to 45 nm) for their antioxidant and anti-inflammatory activities (Bagur et al., 2019). Actinomycete *Streptomyces* are known to produce a broad range of secondary metabolites and can be utilized for the clinical use as antifungals, antibiotics, anticancer, immunosuppressives, antivirals and insecticides (Messaoudi et al., 2015; El-Gamal et al., 2018; El-Moslamy et al., 2018; Singh et al., 2019). *Streptomyces capillispiralis* and *Streptomyces zaomyceticus* Oc-5 have been used for the synthesis of Cu NPs (Hassan et al., 2018; Hassan et al., 2019). Endophytic actinomycete *Isoptericola* SYSU 333150 have been used to synthesized AgNPs (size ranges from 11 to 40 nm) with sunlight exposition using photo-irradiation for different time periods which show antimicrobial, cytotoxic, antioxidant and antiinflamatory effects aginst pathogens (Verma et al., 2016; Singh et al., 2017; El-Gamal et al., 2018; El-Moslamy et al., 2018; Farsi and Farokhi 2018; Abdel-Azeem et al., 2019; Xiaowen et al., 2019; Ranjani et al., 2020). Methods for the characterization of endophytic microorganisms have been illustrated in Figure 6.

Besides endophytic microorganisms, there are several plant species (*Sesbania* plant, *Medicago sativa*, *Brassica juncea*, and *Helianthus annuus*) and microorganisms (bacteria; *Desulfovibrio desulfuricans* NCIMB 8307, *Pseudomonas stuzeri*, *Clostridium thermoaceticum*, *Klebsiella aerogens* and fungi; *Phanerochaete chrysoparium*, *Aspergillus furnigatus*, *Aspergillus flavus*, *F. oxysporum*, and *Verticillium* sp.) that have been used for the synthesis of NPs (Ghormade et al., 2011). In the spinning methods, NPs are synthesized by spinning disc reactor (SDR) (Bhaviripudi et al., 2007; Tai et al., 2007; Mohammadi et al., 2014). The main drawback of CVD is the high-cost related equipment and its highly toxic gaseous by-products (Ealias and Saravanakumar, 2017).

Another important method is pyrolysis for the production of NPs at a large scale. Pyrolysis is a simple, resourceful, low cost, high yield, and constant process. In this method, a precursor (either liquid or vapour) burns with flame and is fed into through a small hole in the furnace at high pressure (Kammler et al., 2001), and the gaseous by-product is characterized to get NPs (Majhi et al., 2018). This green and eco-friendly method for NPs synthesis is called biosynthesis and produces nontoxic and biodegradable NPs using bacteria, plant extracts, and fungi with the precursors (Kuppusamy et al., 2014). This method produces NPs without convention chemicals (Hasan, 2015). Liposomes, vesicles, and micelles are NPs synthesized by supramolecular self-assembly of lipids and surfactants (Vaishnav and Mukherjee, 2019). Basically, micelles are the colloidal aggregates of amphiphilic molecules synthesized using soaps and detergents (Romero and Moya, 2012). Sodium dodecyl sulfate (SDS) and cetyltrimethylammonium bromide (CTAB) are the typical surfactants that form micelles (Hoque et al., 2018). Some lipids and proteins, like lipoxygenase-3, can also aggregate in micelles (26 nm) by heat induction (Brault et al., 2002).

Vesicles/liposomes/lipid vesicles are hollow spheres that are enclosed by amphiphilic molecules (Davies et al., 2006). The vesicles are classified into two types: Unilamellar vesicles (UVs) and Mutilamellar vesicles (MLVs). UVs are defined as having one amphiphile bilayer in the hollow sphere and MLVs are defined as having more than one amphiphile bilayer (Bangham et al., 1965). On the basis of compositions, vesicles are of two types; one is composed of natural or synthetic glycolipids and the other is composed of phospholipids (Romero and Moya, 2012). The properties of having a vesicle-like size and surface potential, polydispersity, degree of ionization, permeability, physical stability, and phase behaviour depend on the methodology used in preparation and the nature of the constituent (da Silva Santos et al., 2019). There are two methods that have been reported for vesicle synthesis spontaneous formation (Jung et al., 2001; Rolland et al., 2004; Segota and Težak, 2006) and vesicle fabrication (Courbin and Panizza, 2004; Segota and Težak, 2006). Spontaneous formation is applied with stress to homogenize the structure without using external energy whereas vesicle fabrication is an induced method to form vesicles *via.* extrusion, sonication, and other methods using external energy. Nowadays, liposomes and vesicles play a significant role in the research field for model systems and permeable biological membranes (Courbin and Panizza, 2004). Some monodispersed branched polymers, such as dendrimers, were found to be different from other linear polymer molecules which can be synthesized through divergent and convergent methods (Hodge, 1993). In divergent methods, two dormant groups and one reactive group-containing a monomer react with a first-generation dendrimer (core-forming) and then successively follows the reaction of several monomers to form large macromolecules. The main drawback of the divergent method is purified form of macromolecule synthesis. Convergent methods rely on the inward synthesis of dendrimers and are easy to purify (Hawker and Fréchet, 1990). Therefore, the development and improvement of novel technology for the synthesis of NPs with their vast applications showed their importance, particularly in the environmental systems and sustainable agricultural (Cheng et al., 2016; Shang et al., 2019; Zehra et al., 2020).

### Nanomaterials as Delivery System

It has been found that NPs play a very significant role as delivery systems in agricultural research for the improvement of crops (Singh et al., 2015). The delivery process of chemicals through NPs in plants is similar to the delivery of nano drugs in humans (Jahangirian et al., 2017; Barupal et al., 2020c). In agriculture, these smart delivery systems should have time-controlled, targeted specific, well-controlled, multifunctional characteristics, and should be self-regulated to evade biological barriers. Plants and their extracts have been used to synthesize several NPs and were found to be more ecofriendly with specific well-defined size and shapes (Agarwal et al., 2017; Ahmed et al., 2017; Meena and Zehra, 2019). NPs as delivery systems have been applied in agricultural applications for the improvement of crops by studying their effect on plant growth, metabolic functions, and genetic transformation. Nano-encapsulated chemicals for agricultural purposes should be planned in a manner to show less ecotoxicity, effective concentration, high stability, solublility, time-control, and to enhance their targeted activity when certain stimuli occur (Mathur, 2016). Perez-de-Luque and Rubiales (2009) reported that nanocapsulated herbicides reduce the phytotoxicity caused by herbicides under parasitic weed control.

These nanocapsules have the ability to penetrate cuticles and release active ingredients to control target weeds. The diameter of NPs should be less than the diameter of a plant’s cell wall (5–20 nm) to penetrate and reach the plasma membrane (Schwab et al., 2016). NPs can enter into the plant cell through stomatal openings or bases of trichomes, and are then translocated to tissues (Nair et al., 2010). Lipophilic nanosilica get easily absorbed into the cuticular lipids (effective barrier made of several lipids and fatty acids) of insects through physiosorption process and destroy the protective wax layer for use as pest control in agriculture (Jampílek and Kráľová, 2017). Ag-NPs (1–5 nm) have been successfully used to control phytopathogens (Surega et al., 2019). Currently, nanotechnology applications have been employed to study biological systems in medical research and animal science.

The use of nanotechnology and their versatility can also be demonstrated in plant science research to study genomics and the function of genes for the improvement of crop species. It has been shown that silica NPs can be used to deliver drugs (Giri et al., 2005) and DNA material (Bharali et al., 2005; Meena and Samal, 2019) into animal cells and tissues but their delivery into plant cells is limited due to the presence of a cell wall. 3 nm pores containing mesoporous silica nanoparticles (MSN) can transport chemicals and DNA into plant cells due to having exclusive structural features, such as their thermally and chemically stable mesoporous structures. Mesoporous structures have well-defined surface properties with pore sizes (2–10 nm in diameter) and surface areas more than 800 m^2^ g^−1^, and are preferred as an ideal host for the various properties containing guest molecules. In most of the non-porous Au or Ag coated particle-based (such as in gene gun process) DNA or chemical delivery limitations were shown to the nucleic acid. The microinjection process can also be used for DNA delivery to the plant cell for genetic modification but they were found to be inefficient (Ahmad and Mukhtar, 2017; Meena et al., 2017g; Meena et al., 2017h). The specific feature of MSN is to prevent the leaching out of loaded molecules or drugs due to covalent bonding with the pore. The molecules are released by some chemicals (uncapping triggers), such as dithiothreitol (DTT), or disulphide-reducing antioxidant inside the cells (Torney et al., 2007). The interactions of these capped MSN systems have been studied in plant cells (without cell wall) compared with animal cells. In animal cells, endocytosis is a very efficient process as compared to plant cells due to membrane impermeability (Jat et al., 2020). Torney et al. (2007) reported that endocytotic vesicles size ranged between 0.2 and 3 mm and showed no toxicity to plants cells.

The mesoporous structure of the MSNs enables the delivery of those chemicals which are incompatible with growth media and impermeable to the membrane along with DNA material to the targeted cells. Further, developments like enlargement of pore size and more functionality of these MSNs will offer new potential and possibilities in the delivery of target specific proteins, chemicals, and nucleotides in plant biotechnology. Overall, the MSN system appeared as a new and versatile tool to study cell biology and plant endocytosis. Each plant has a specific defense mechanism to protect itself against phytopathogens and herbivorous insects. This defense mechanism is further translated into a suitable adaptive response to defend against pathogen attack (Dangl and Jones, 2001; Barupal et al., 2019). This mechanism either can be triggered or activated after a pathogen attack or be pre-existing (Koornneef and Pieterse, 2008). Under pathogen attack, plants showed resistance against pathogens which is referred to as induced systemic resistance (ISR). ISR is the alternative natural and clean biological process of an integrated pest management strategy to control plant diseases (Sticher et al., 1997; Van Loon, 1997). In cucumber plants, SiO_2_ NPs reduced the infection of papaya ring spot virus (PRSV) by inducing certain defense-related gene expressions to activate phenylalanine ammonia-lyase (PAL) genes peroxidases (POX). Most of the important food crops are affected by bacterial wilt which is a serious problem. Recent studies have indicated the resistance of plants to bacterial infection can be increased by treatment with biotic or abiotic stress factors. MgO-NPs showed disease resistance in tomato plants against *Ralstonia solanacearum*. MgO NPs significantly reduced the bacterial (*R. solanacearum*) infection in the root of tomato seedlings by inducing rapid synthesis of reactive oxygen species (ROS) such as oxygen-free radicals.

It has been clearly investigated that NPs induce defense-related mechanisms against pathogens through ISR. NPs such as chitosan biopolymer are biocompatible, biodegradable, and non-toxic in character and therefore can be used as delivery systems for micronutrients and immune elicitors to suppress disease in plants (Kumaraswamy et al., 2018; Meena et al., 2017c; Meena et al., 2017d). Cu and Zn CS-NPs (chitosan NPs) were synthesized by entrapping metal in chitosan. These CS-NPs were useful for controlling plant diseases like Curvularia leaf spot (CLS) of maize (Choudhary et al., 2017) and blast disease of finger millet (Sathiyabama and Manikandan, 2018) by inhibiting mycelial growth of pathogens and activating the plant growth. CLS controlled by 0.04–0.16% CS-NPs about 24.6–22.6% in the pot while in 44.0% of water condition (Choudhary et al., 2017). Cu-CS-NPs-treated plants showed 11.6% enhancement in grain weight as compared to Bavistin treated wheat plant similar to the case of Zn-CNPs. Higher concentrations of Cu-CNPs and Zn-CNPs negatively affect plant growth (Fu et al., 2020). These CS-NPs release their metals Cu^2+^ and Zn^2+^ to interact with the cellular system of plants and facilitate other metabolic processes of plants based on Cu and Zn nutrition (Rajasekaran and Santra, 2015; Saharan and Pal, 2016). Therefore, Cu-CNPs and Zn-CS-NPs improve the plant growth as well as protect from phytopathogens as plant immune elicitors by showing multimodal action. CS-NPs have emerged as better immune elicitors as compared to salicylic acid (SA) and harpin (Thakur and Sohal, 2013). The harpin_Pss_-loaded CS-NPs (H-CS-NPs) improved the damping-off in tomato caused by a phytopathogen *Rhizoctonia solani* (Nadendla et al., 2018). SA functionalized chitosan nanoparticles (CS-NPs) control the *Fusarium verticillioides* causing post-flowering stalk rot (Kumaraswamy et al., 2019) and showed strong antifungal activity by growth inhibition of mycelia 62.2–100% at 0.08–0.16% of CS-NPs. Therefore, present studies showed strong evidence regarding how NPs act as an efficient delivery system for the continued release of bioactive compounds that trigger the plant immune system to enhance the long-lasting effect of disease suppression efficacy. NPs also work as biostimulants at a specific concentration and play a very important role in disease suppression in plants.

### A Brief Discussion of Engineered Nanomaterials

For the specific physical and chemical properties, nanomaterials are engineered at 1–100 nm in particle size (Wilson et al., 2002). These engineered nanomaterials are nanoscale metals and contain oxides (e.g., iron oxides, aluminum oxides, and titanium dioxide), polymeric nanocomposite materials and polymers. Engineered nanomaterials have also been used in drug delivery, immunology, and photovoltaic cells. (Bhatia, 2016). Engineered nanomaterials play a very important role in energy generation, production of food (Morris, 2011), and remediation of water to remove toxic substances or pollutants (Hochella et al., 2019). Currently, due to having a large surface area and small size, engineered nanomaterials are discussed to improve plant growth and health with good soil quality for sustainable agriculture. These engineered nanomaterials are highly potent for soil feasibility in soils due to being highly reactive and containing distinct properties such as high cation exchange capacity, longlasting release of nutrients, and delivery of nutrients to solve the problem of soil restoration (Bastioli, 2020). Metallic oxide-based NPs such as Mn, Fe, Cu, and Ag have been widely used in biological processes (Amde et al., 2017).

In another aspect, the synthesis and use of massively engineered nanoproducts released into the environment interact with several components of the environment and are followed by dynamic transformation processes (Abbas et al., 2020). These transformations of engineered NPs are interrelated to several environmental aspects. Several environmental processes, such as physical, chemical and biological changes the mobility and availability of these engineered NPs. Physico-chemical features of engineered NPs and environmental factors (pH, temperature, ionic strength, organic, inorganic colloids, etc.) are very important conditions to transform engineered NPs (Goswami et al., 2017). Therefore, it is of high importance to study the activities of transformed engineered NPs to recognize their environmental fortune, bioavailability, and form of toxicity. Some toxicological studies revealed that some freely circulating engineered NPs can be toxic for living systems. They affect the capability and behaviour of the plants (Aslani et al., 2014).

### Interaction of Nanoparticles With Plants

**FIGURE 7:**
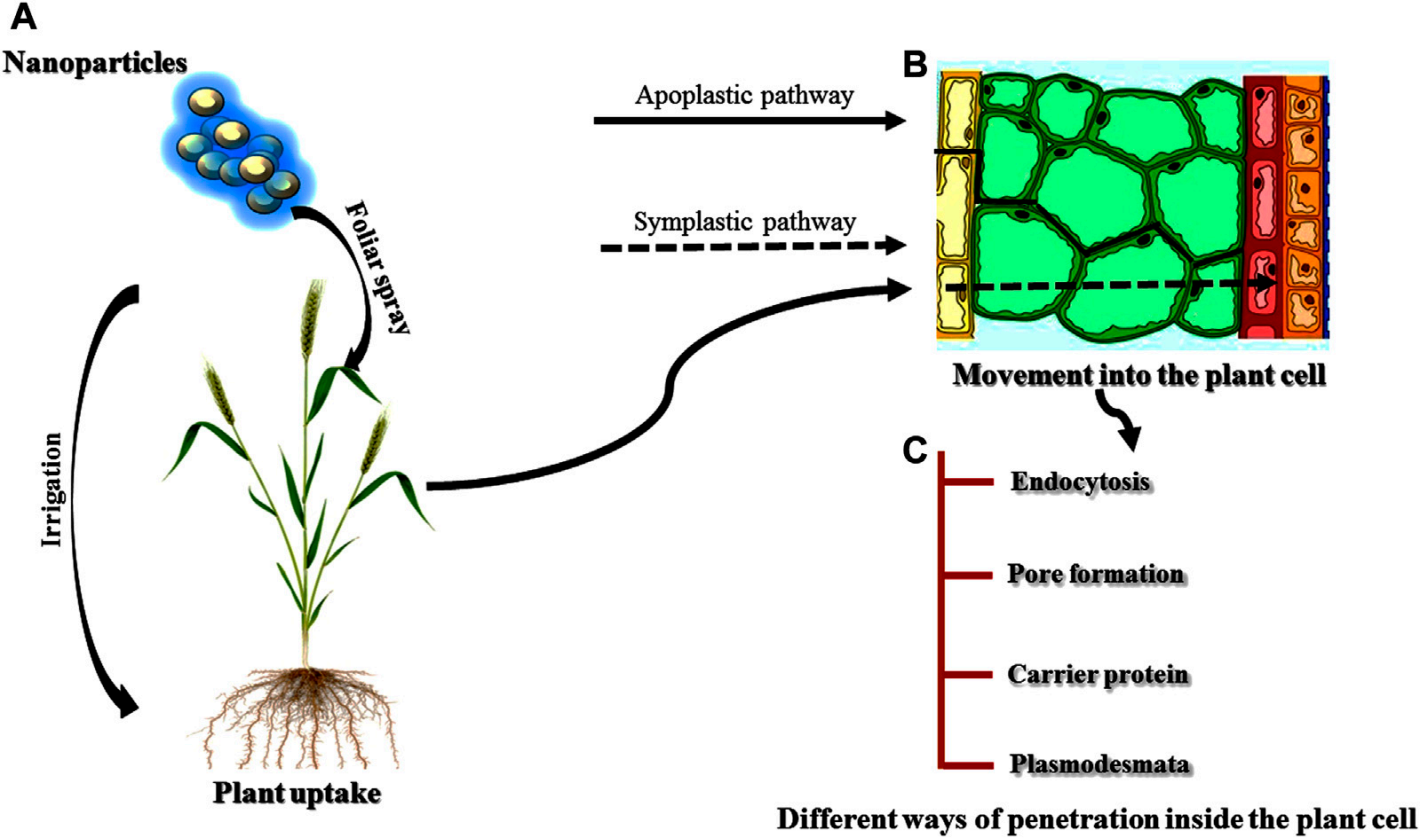
**(A)-(C)** Uptake, movement and penetration of nanoparticles inside the plant cell.

Nanoparticles (NPs) due to their various properties are being used in the fields of biotechnology and agriculture (Pérez-de-Luque, 2017). Different factors such as the nature of the NPs, plant physiology and interaction of the NPs govern the uptake of NPs by the plants (Khan M. R. et al., 2019; Figure 7A-C). Chemical entities, stability, and functionalization of NPs influence the uptake, translocation, and accumulation; properties are also found to be variably affected by plant type, species, and site facilitating internalization of NPs (Santana et al., 2020). Different studies have reported both the positive and negative effects of the NPs on the plants (Yang et al., 2017; Goswami et al., 2019; Kumar et al., 2019). Zinc oxide NPs showed a positive effect on soybean by increasing its root length whereas negative effects (shrunken root tip and broken root caps) were found in ryegrass (Lin and Xing, 2007; López-Moreno et al., 2010; Meena et al., 2017e; Meena et al., 2017f). Similarly, Cañas et al. (2008) also reported both positive and negative impacts of single-walled carbon nanotubes (SWCNTs) in root length of onion and cucumber, respectively.

### Absorption, Uptake, and Translocation of Nanoparticles by the Plants

It is well demonstrated that the properties of NPs are the main factor in absorption by the plants (Khan I. et al., 2019; Santana et al., 2020). Among the different properties of NPs, size of the NPs is one of the main factors affecting the penetration, translocation, and accumulation of NPs to the plant cells (Lv et al., 2019). NPs with a size larger than 40–50 nm are restricted by the plant cells for absorption (Avellan et al., 2017; Pérez-de-Luque, 2017). Chemical composition, morphology, and the type of NPs are other factors affecting the uptake and translocation (Elemike et al., 2019; Sanzari et al., 2019). Additionally, Judy et al. (2012), studied another factor responsible for absorption and accumulation i.e. functionalization and coating of the nanomaterial surface. The absorption and accumulation of NPs by the plants are greatly affected by the functionalization and coating. Different researchers revealed that the physiology of plants is also an important factor influencing the uptake and translocation of NPs (Pérez-de-Luque, 2017; Khan M. R. et al., 2019). Some NPs exposed to different plant species belonging to different families showed different absorption and accumulation patterns in the plants (Balafrej et al., 2020). The effectiveness of penetration to the plant cells is greatly determined by the application method of NPs because roots and leaves are both specialized in different processes (Schwab et al., 2016).

The interactions of NPs with the environment also affect the properties of NPs, and in turn influence the uptake of NPs by the plants. Navarro et al. (2008) studied the effect of organic matter and salt ion on the absorption of NPs and found that the stability of organic matter provided better availability of the NPs to the plants whereas salt ions showed opposite results. Microbes present in the soil also influence the uptake of NPs to the plants especially mycorrhizal fungi. Mycorrhiza forms a symbiotic association with the roots of plants and hence provides a better platform for the NPs to get easily absorbed by the plants (Ingle et al., 2017; Cao et al., 2020). Once the NPs are absorbed by the plants, the translocation of the NPs is achieved in two different ways: the symplast and the apoplast (Meena et al., 2017i; Lv et al., 2019). The transport of NPs *via.* apoplastic pathways occur through extracellular spaces and cell walls of neighbouring cells and xylem vessels (Sanzari et al., 2019) whereas symplastic transport takes place through plasmodesmata between the two adjacent cells (Ruttkay-Nedecky et al., 2017) and sieve plates. The importance of apoplastic pathways is very crucial for the movement of the NPs within the plants (Schwab et al., 2016). NPs reach the central cylinder and vascular tissue of the roots *via.* this pathway and further move to the aerial parts of the plants through the xylem with the help of transpiration stream (Cifuentes et al., 2010; Banerjee et al., 2019).

Still, there is a barrier to reaching the xylem of NPs through the root *via.* the apoplastic pathway called the Casparian strip which can be overcome by the endodermal cells following the symplastic pathways. Different studies have reported the accumulation of some NPs at the Casparian strip (Schwab et al., 2016; Rossi et al., 2017). Translocation of NPs *via.* the symplastic pathway through the sieve tube elements of the phloem allows the distribution of NPs toward non-photosynthetic tissues and organs (Shukla et al., 2016; Banerjee et al., 2019). Foliar application of NPs involves the crossing of the cuticle which acts as a barrier for the NPs following the lipophilic or hydrophilic pathways (Avellan et al., 2019). Hydrophilic and lipophilic pathways involve diffusion through cuticular waxes and through the polar aqueous pores present in the cuticle and/or stomata (Fernández and Eichert, 2009; Fernández et al., 2017). In the case of foliar application, the stomatal pathway is the main route for the interaction of NPs above 10 nm. The tiny size of the cuticular pore (around 2 nm) makes it less efficient for the translocation of NPs (Eichert and Goldbach, 2008).

The information about the accumulation of NPs inside the plants mainly depends on the route of translocation (Singh J. et al., 2018). For example, if a kind of NP shows a good translocation through the xylem, application should be done to the roots, whereas if the main route of any NPs is the phloem, not xylem so they should be applied by foliar spray for the even distribution of the NPs. If the route of the translocation of the NPs is known, the accumulation of NPs in plant parts can be found. For example, if any NP is translocated through the phloem, it must be accumulated in fruits and grains. However, it is not necessary that translocation will takes place with a specific cell. Lateral movement of NPs between the xylem and phloem can also occur (Pérez-de-Luque, 2017). Translocation and accumulation of the NPs are greatly influenced by the characteristics and nature of the NPs, in addition to the physiology of the plant species (Remédios et al., 2012; Lv et al., 2019). Different studies have reported the differences in the mechanism of translocation and accumulation of the same kind of NPs for different plant species (Shang et al., 2019; Yan and Chen, 2019; Hossain et al., 2020). On the contrary, similar NPs with few differences showed different results within the same plant species (Zhang P. et al., 2019). In pea plants, faster translocation and large accumulation of carbon-coated iron NPs were found in the roots whereas slow translocation and less accumulation of the same NPs were reported in sunflower and wheat (Cifuentes et al., 2010). Further, a large amount of positively charged gold NPs were accumulated by radish and ryegrass than rice and pumpkin (Zhu et al., 2012). Negatively charged Au-NPs were not taken up faster by the roots of the plants because plant cell walls contain negative charges resulting in the accumulation of positively charged Au-NPs.

NPs generally accumulate to different plant parts, such as fruits (McClements and Xiao, 2017), grains (Mahakham et al., 2017), flowers, and young leaves (Padalia et al., 2015; Javed et al., 2019) after the translocation through the vascular system. The location of the accumulation of NPs within the plants can be crucial to avoid human and animal consumption of NPs after treatment. Different studies have demonstrated the storage of NPs in the plant parts which are not used for consumption and degradation or transformation of some NPs by the plant after some time (Kalpana and Devi Rajeswari, 2018; Khan I. et al., 2019; Salem and Fouda, 2020). Higher concentrations of NPs affects human health. Human exposure to NPs takes place *via.* three different routes-gastrointestinal, skin and lungs and is then distributed to the blood and brain after absorption and subsequently, to heart and kidney (Korani et al., 2015).

### Interaction of Nanomaterials With Plant Cells

If the NPs are to be translocated by the symplastic pathway, they must be taken by the plant cell and cross the plasma membrane (Karny et al., 2018). There are different ways for the internalization of the NPs to take place. Nanoparticles can be taken by the plant cell through the process of endocytosis and can cross the plasma membrane (Etxeberria et al., 2016). Some NPs instead of being invaginated by the plasma membrane are taken up by the cell by the formation of pores on the plasma membrane which directly reaches the cytoplasm (Behzadi et al., 2017; Zhao and Stenzel, 2018). NPs can also bind to carrier proteins of the plasma membrane that internalize the NPs inside the plant cell (Lesniak et al., 2013). Several researchers have acknowledged aquaporins as the carrier protein for internalization of the NPs to the plant cells, however the tiny pore size creates a hinderance for NP penetration (Banerjee et al., 2019), without reorganisation and enhancement of pore size. Plasmodesmata are very important structures of plant cells for the translocation of NPs through the phloem (Fincheira et al., 2020). Additionally, ion channels are also used by the NPs for entry into the plant cells but the tiny size of the channels makes it not suitable for the NPs penetration without specific modifications (Chichiricco and Poma, 2015; Pérez-de-Luque, 2017). Endocytosis appeared to be the most suitable way for the delivery of chemicals inside specific cell organelles (Iversen et al., 2011). On the other hand, pore formation is the best way for the delivery of chemicals into the cytosol.

## Molecular Approaches of Nanoparticles

### Gene Carriers

It has been observed that an effortless DNA conveyance strategy would encourage investigations of plant functional genomics (Rai et al., 2015). Nonetheless, the effect of NPs on plants is limited by the plant cell wall (Torney et al., 2007). There are different relevant properties of NPs with the ability to cross biological membranes, carry out intracellular multifaceted target delivery, and perform controlled release having enabled NPs to revolutionize the genetic engineering method (Cunningham et al., 2018). However, plant cell walls act as a barrier for efficient nanocarrier delivery which is generally conquered by chemical or mechanical methods (Demirer et al., 2017). DNA and chemicals were first delivered by Torney et al. (2007) to tobacco plants through biolistic delivery of 100–200 nm gold-capped MSNs. In this method, Gold NPs were capped by the MSN pores which were loaded with the chemical expression inducer. The coating of green fluorescent protein (GFP) plasmids was done to the capped MSNs and delivered to the tobacco cotyledons by gene gun. Thereafter, unsealing and release of the chemical expression inducer caused the expression of GFP. This study demonstrated the proof role of NPs as a gene carrier into the plant cells. In addition to this, Martin-Ortigosa et al. (2014) reported the delivery of Cre recombinase proteins into the *Zea mays* cells using the gold plated MSNs by the biolistic method. Different strategies comprising of gene gun, electromagnetic field, and protoplast polyethylene glycol transfection are still mandatory for the efficient delivery of biomolecules into the plant cells by NPs, as NPs cannot passively bypass the plant cell wall (Cunningham et al., 2018; Lu, 2018; Rastogi et al., 2019). Even after the requirement of mechanical and chemical aid for internalization of NPs, nanocarriers still show superior performance over traditional methods because of their small size and high surface area (Shang et al., 2019). Several studies have demonstrated the successful mediated delivery of NPs to the plants *in vivo* (Raliya et al., 2016; Zhao et al., 2016; Lee et al., 2017) and *in vitro* (Pasupathy et al., 2008; Naqvi et al., 2012; Burlaka et al., 2015). Chang et al. (2013) performed fluorescence and antibody labelling techniques for the detection of gene expression in the epidermal and endodermal layer of *Arabidopsis thaliana* roots by using MSNs as a gene carrier to deliver foreign DNA into the plants.

Moreover, Demirer et al. (2018) studied the efficient delivery of plasmid DNA and siRNA into *Eruca sativa* and *Nicotiana benthamiana* plants using functionalized carbon nanotubes (CNT) NPs. In the leaves of *E. sativa*, the green fluorescent protein (GFP) was expressed whereas expressed GFP was silenced in transgenic *Nicotiana benthamiana* leaves. Further examinations are expected to advance NP properties and functionalization, since early outcomes are promising for additional investigation of NPs as a plant biomolecule delivery vehicle that tends to the drawbacks of the traditional strategies. This could work alongside with the appearance of nuclease-based gene-altering advancements. It is of incredible interest to researchers to improve the delivery of these progressive genome designing tools by investigating NP-based delivery techniques for assorted biomolecular cargoes.

### Genetic Modification

The genetic modification of plants has been broadly investigated for the production of new varieties of crop plants with several desirable characters such as high yield, improved quality, and resistance against abiotic and biotic stress (Kumar et al., 2020). Practically, tissue culture is the main technique used in almost all of the current strategies of genetic engineering, although they are very tedious, long, and relentless procedures (Zhang et al., 2020). It is very difficult for some of the agriculturally important crop plants, such as cotton to produce transgenic plants from the tissue culture with conventional plant breeding methods. So, there should be an alternative method to overcome the constraints of traditional tissue culture methods and its associated problems. Pollen-based plant transformations are viewed as promising alternatives over traditional methods of transformation (Zhang R. et al., 2019). During pollination and fertilization, foreign DNA is directly released to the ovary by pollen grains. There is a direct production of transgenic seeds with foreign DNA transformed pollen by the process of pollination. Different physical methods such as electroporation, bombardment, sonication, and *Agrobacterium* infection have been used for the transformation of pollen, however its success rate is restricted. Although, this technique is promising, they are also unfavorable to pollen viability. An ideal and highly efficient method of pollen transformation is magnetofection in which a foreign DNA associated with magnetic NPs is adroitly taken up by the target cells of pollen in the presence of a magnetic field (Zhao P. et al., 2017). One of the molecular approaches of NPs is genetic modification. Pollen magnetofection is the genetic modification of pollen using NPs.

In this technique, pollens are genetically transformed with the help of magnetic NPs which are loaded with pure plasmid DNA carrying functional genes. Pure plasmid DNA is delivered into the pollen through a pollen aperture in the presence of a magnetic field. Genetically modified pollen (magnetofected pollens) produces transformed seeds through pollination (Bisen et al., 2015; Zhang R. et al., 2019). One of the main advantages of this technique is that foreign DNA can stably express in successive generations. Pollen magnetofection is an effective stage for genetic modification of cotton and other crops with high-throughput and proficient potential infield activity (Altindal and Altindal, 2020). The wall of pollen is reduced at the surface apertures with a diameter of about 5–10 μm in most of the crop pollens. Zhao P. et al. (2017) reported the presence of such aperture in cotton pollen where the wall of pollen was thin with high permeability. The thin pollen wall made the delivery of foreign DNA possible inside the pollen. MNPs were used by Zhao P. et al. (2017) as DNA carriers that could easily pass through the apertures under the influence of a magnetic field. In pollen magnetofection, an MNP-DNA complex was formed by binding and condensing the negatively charged DNA with the positively charged polyethyleneimine-coated Fe_3_O_4_ MNPs which inconsequentially acts as a DNA carrier. Then, pollen was mixed with MNP–DNA complexes. Subsequent mixing of the MNP-DNA complexes were directed into the pollen through pollen aperture under the influence of the magnetic field before pollination. After the formation of the transformed seeds, transgenic plants were obtained by kanamycin screening.

### RNA Interference

The RNAi pathway has risen as an amazing asset to battle plant pathogenic microbes by genetic engineering (Robinson et al., 2014; Majumdar et al., 2017). Effective use of dsRNA has developed as a profoundly engaging alternative. Up till now, nanocarriers of RNAi-inducing molecules have been used against viruses, aphids, and mosquitoes (Das et al., 2015; Mitter et al., 2017; Thairu et al., 2017). Silva et al. (2010) reported the knockdown of a target gene in tobacco protoplasts through encapsulation of siRNAs into conjugated polymer NPs. Draz et al. (2014) reviewed the use of different NPs such as metal and metal oxides NPs, silica and silicon-based NPs, carbon nanotubes, dendrimers, graphene, polymers, cyclodextrins, lipids, semiconductor nanocrystals, and hydrogels as a carrier for dsRNA. In the seedlings of *Arabidopsis*, fluorescent NPs loaded with dsRNA induced the gene silencing of two endogenous genes (Jiang et al., 2014). Mitter and colleagues sprayed the plants with Bioclay, a layered double hydroxide (LDH) NP loaded with dsRNA against the two viruses *viz.* pepper mild mottle virus (PMmoV)and cucumber mosaic virus (CMV) (Mitter et al., 2017). Further, Worrall et al. (2019), synthesized BCMVCP-BioClay by the encapsulation of BCMVCP-dsRNA (which targets the coat protein (CP) coding region of bean common mosaic virus) into LDH-NPs and reported the enhanced protection of *Nicotiana benthamiana* and *Vigna unguiculata* against aphid-mediated virus transmission as compared to the naked dsRNA. However, even though exogenous use of RNAi-inducing molecules for crop improvement still has advantages over pesticides, because of its decreased toxicity, effective use of RNAi still faces its own obstacles.

### Application of Nanoparticles

#### Applications of Nanotechnology to Increase the Production Rate and Crop Yield

Different methods such as plant breeding, fertilizers, and plant protection products have been used for increasing the crop yield (Usman et al., 2020). The decline in agricultural productivity has been reported since the green revolution which needs another revolution in agricultural technology (Ghidan and Al Antary, 2019). Nanotechnology is a quickly emerging field with the possibility to advance forward the agriculture and food industry with new devices and tools which guarantee to increase food production in a sustainable manner and to protect crops from various diseases (Moulick et al., 2020). The management of the primary production of crops highly depends on two main fundamental aspects: increased crop production and nutrient use efficiency (Usman et al., 2020). Nanofertilizers and nanobionics both meet these two aspects and play important roles in agriculture by increasing the production rate and crop yield (Shang et al., 2019).

### Nanofertilizers

The consistently growing human population is creating pressure for the agriculture sector to fulfill their continuously increasing demands (Zulfiqar et al., 2019). Chemical fertilizers that are generally used for improving crop productivity have major adverse environmental and ecological effects (Pirzadah et al., 2020). Nanotechnology which utilizes the small size of NPs (less than 100 nm) with unique properties such as higher absorption rate, utilization efficacy, and minimum losses may offer an exceptional opportunity to create a concentrated source of plant supplements (Iqbal, 2019). Nanofertilizers are being synthesized by encapsulating the plant nutrients into nanomaterials and delivering them in the form of nano-sized emulsions (Kah, 2015). The uptake and deep penetration of nanomaterials are facilitated by the nanopores and stomatal openings in plant leaves leading to higher nutrient use efficiency. Plasmodesmata which are nanosized channels between cells facilitate higher transport and delivery of nanofertilizers (Pirzadah et al., 2020). The increased efficiency of utilization causes significantly less nutrient losses of nanofertilizers which ultimately leads to higher productivity and nutritional quality of various crops.

Different approaches, such as top-down, bottom-up and biological methods (especially endophytic), are generally used for the synthesis of NPs as nanofertilizers (Shang et al., 2019; Messaoudi and Bendahou, 2020). Nanofertilizers are generally of two types, macronutrient nanofertilizers and micronutrient nanofertilizers. Different macronutrients such as nitrogen, phosphorus, potassium, magnesium, sulphur and calcium encapsulated with NPs reduce their overall requirements and deliver precise amount of nutrients to the crops (Zulfiqar et al., 2019). Nanofertilizers consist of one or more macronutrients with specific NPs. Nanofertilizers such as zeolites, hydroxyapatite and mesoporous silica NPs containing nitrogen macronutrient have been reported to show promising results by increasing the production and yield in different food crops (Fatima et al., 2020). Nanofertilizers are also synthesized by encapsulating the micronutrients to meet the requirements of different crop plants. Zinc (Zn) plays a very important role in plant growth by acting as a regulatory cofactor for various enzymes (Umair Hassan et al., 2020). Zinc has also been reported to provide protection to the plants against different pathogens (Cabot et al., 2019). Boron is also very important for the growth and development of plants as it is involved in the biosynthesis of the cell wall and its lignifications (Wimmer et al., 2019). Hence, it is crucial to apply the appropriate amount of Zn and B to different food crops for higher yield and good quality. Davarpanah et al. (2016) studied the effect of three different concentrations of nanofertilizers of Zn and B on the yield and quality of pomegranate and observed that the maximum fruit yield along with good quality was improved by the application of low amounts of B and Zn. In another study, the fruit yield and growth of shoots was increased in cucumber seedlings grown in nutrient solution containing rubber type NPs as Zn source as compared to commercial Zn sulphate fertilizer (Moghaddasi et al., 2013).

Further, Tarafdar et al. (2014) developed zinc nanofertilizers for the enhancement of crop production in pearl millet (*Pennisetum glaucum* L.) and found that the growth and yield of the crop were significantly enhanced by the use of zinc nanofertilizers. Several studies have reported the effect of different nanofertilizers on increased crop production in many cereals (Jyothi and Hebsur, 2017). Maghemite NPs improve crop production and stress tolerance by reducing the hydrogen peroxide content as well as lipid peroxidation in *Brassica napus* plants (Palmqvist et al., 2017). Fe is also a very important micronutrient for the growth and development of plants. Hu et al. (2017) studied the effect of different concentrations of iron oxide NPs and ferric ions on the physiological changes in *Citrus maxima* plants and demonstrated that the effect of nanofertilizers on plants was different at different concentrations. At very low concentrations there was no effect on the plants whereas at very high concentrations, plants were negatively influenced. This suggests that the effect of iron oxide NPs was concentration-dependent. Manganese (Mn) also plays an important role in various physiological processes by acting as a cofactor of various enzymes. Stabilized NPs of copper, zinc, manganese, and iron oxide NPs showed different effects on lettuce seedlings. Mn and Fe NPs enhanced plant growth whereas CuO NPs were more toxic than the Cu ions. The toxicity of ZnO NPs was similar to Zn ions (Liu et al., 2016).

### Nanobionics

Plant nanobionics is a combination of plant biology and nanotechnology and it deals with the enhancement of plant productivity by improving plant growth development and photosynthetic efficiency (Sharma and Kar, 2019; Ansari et al., 2020). Nanobionics use nanomaterials for the enhancement of plant productivity (Lew et al., 2020). Photosynthetic efficiency can be improved by widening the range of solar light absorption near-infrared spectra. Nanomaterials with unique properties and higher stability can form chloroplast based photocatalytic complexes with enhanced and improved functional properties (Marchiol, 2018). Different studies have reported on the positive effects of nanomaterials on photosynthesis (Qi et al., 2013; Giraldo et al., 2014). The high photocatalytic activity of titanium oxide nanoparticles (nTiO2) play a role in the enhancement of absorption of light by the leaves and increase photosynthesis. nTiO_2_ enhances the photosynthetic rate by influencing the electron chain transport, photophosphorylation activity, Rubisco carboxylation, and protection of chloroplast from ageing (Linglan et al., 2008; Qi et al., 2013). It also positively influences water conductance and transpiration. Giraldo et al. (2014) studied the effect of single-walled carbon nanotubes (SWCNTs) on the photosynthesis process in leaves of *Arabidopsis thaliana* and isolated chloroplasts of *Spinacia oleracea*. The authors observed that the shelf life of isolated chloroplast and electron transport rate was highly increased in the treated leaves and chloroplast. The advantage of semiconductor SWCNTs over chloroplasts was having high electrical conductance and the ability to capture solar energy in wavelengths that were weakly absorbed by chloroplasts. Three times higher photosynthetic activity and enhanced electron transport rate were promoted by the SWCNT-chloroplast assemblies than control (Giraldo et al., 2014). From one perspective, there is no uncertainty that further comprehensive research would be expected to assess the impacts of plant nanobionics on enhanced production of sugars as well as crop yield. Then again, the upgrade of a fundamental plant function because of the consolidation of nanomaterials was shown as confirmation of the concept.

## Role of Nanotechnology in Crop Protection

### Antimicrobial Agents

**FIGURE 8:**
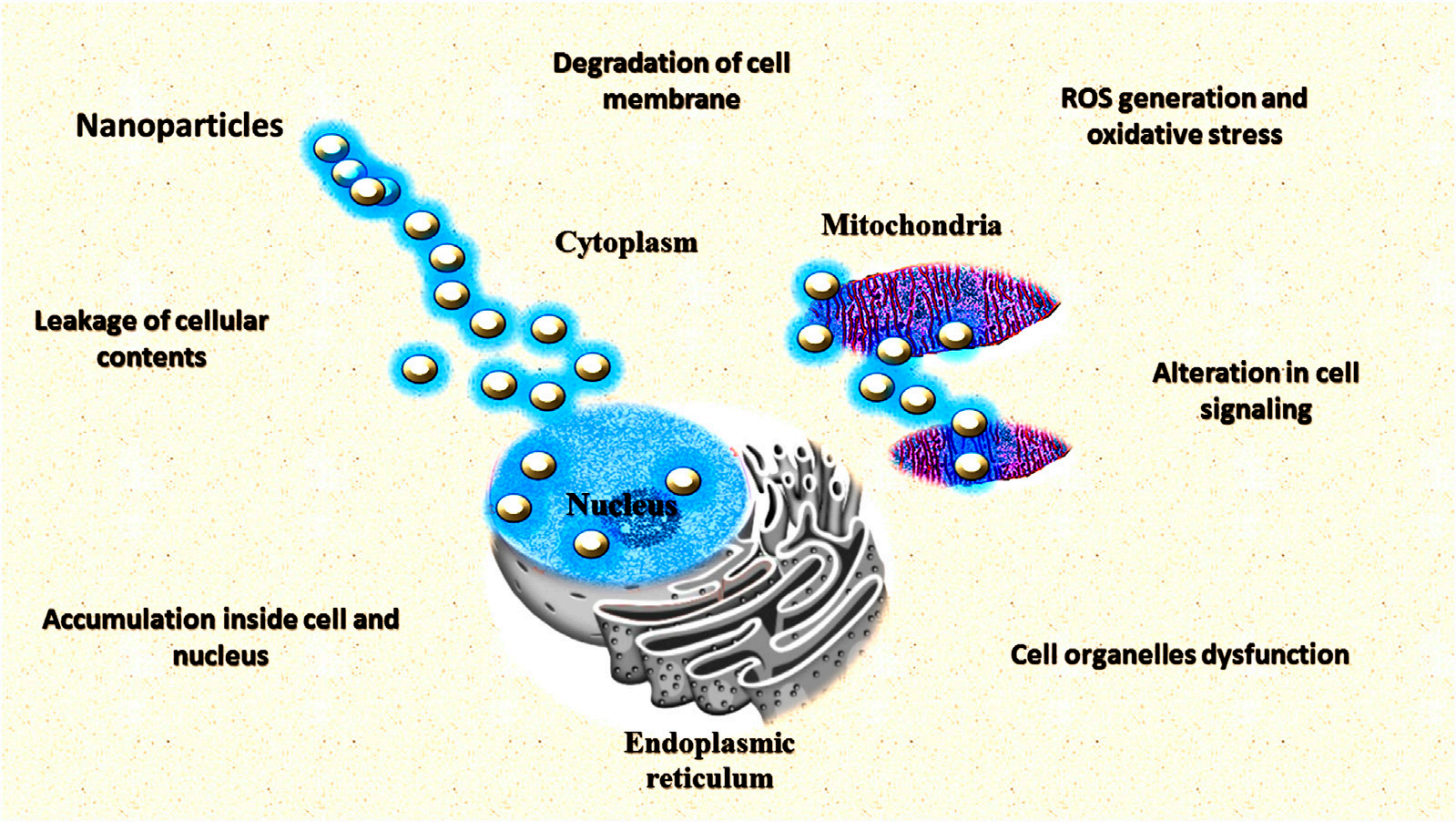
Different mechanisms of nanoparticles as antimicrobial agents.

Nanoparticles are one of the most promising agents to prevent the emergence of antimicrobial resistance against pathogenic microbes such as *Fusarium oxysporum*, *Alternaria solani, Aspergillus niger*, *Ralstonia solanacearum*, and *Erwinia amylovora* (Wang Y. et al., 2017; El-Batal et al., 2020). Acording to Chavan and Nadanathangam (2019), the use of higher concentrations of Ag and ZnO NPs (3,000 μg/ml) affect the three groups of agriculturally relevant beneficial microorganisms. The exclusive physiochemical properties of NPs and growth inhibition of pathogens make it a potential candidate for antimicrobials (Karaman et al., 2017). Different metals such as silver and copper have long been used for treatment against pathogenic microbes. It is very obvious that some of the metallic compounds have antimicrobial properties. Lately, some of the metals in the form of NPs have been used as promising antimicrobial agents. Various kinds of metallic NPs *viz.* aluminium, copper, gold, magnesium, silver, titanium, and zinc NPs are found to have antimicrobial properties (Sánchez-López et al., 2020; Table 1). Different NPs inhibit microbial growth through different mechanisms (Figure 8).

**TABLE 1:** Antimicrobial activities of different nanoparticles.

Nanoparticles	Methods of synthesis		Target organism	Mechanism of action		References
**Antibacterial activity**							
Silver nanoparticle	Immersion method		*Escherichia coli*, *Strptomyces aureus*, *Bacillus subtilis*, *Staphylococci*, *Pseudomonas aeruginosa*	Generation of ROS, Degradation of cell membrane, Leakage of cellular contents, Interaction with phosphorus moieties in DNA resulting in inactivation of DNA replication, Reaction with sulfur-containing amino acids leading to the inhibition of enzyme functions		Lala et al. (2007), Andrade et al. (2016), Khatoon et al. (2017)
Gold nanoparticle	Immersion method		*B. subtilis*, *E. coli*, *Klebsiella mobilis*, *Staphylococcus aureus*			Zhang et al. (2008), Rai et al. (2010)
Copper oxide nanoparticle	Gel combustion method		*E. coli*, *P. aeruginosa*, *Staphylococcus aureus*, *B. subtilis*			Ren et al. (2009), Azam et al. (2012b)
Zinc oxide nanoparticle	Green synthesis		*E. coli*, *Salmonella enteritidis*, *B. subtilis*, *Staphylococcus aureus*, *Proteus mirabilis*, *Serratia marcescens*			Jin et al. (2009), Gunalan et al. (2012), Singh V. P. et al. (2018)
Magnesium oxide nanoparticle	Aerogel method		*E. coli*, *B. subtilis*, *Bacillus megaterium*			Richards et al. (2000), Koper et al. (2002)
Aluminum oxide nanoparticle	Immersion method		*E. coli*			Li and Logan, (2004)
Titanium dioxide nanoparticle	Batch technique		*E. coli*, *Staphylococcus aureus*, *Lysteria monocystogenes*			Hu et al. (2006), Chawengkijwa-nich and Hayata (2008)
**Antifungal activity**							
Silver nanoparticle	Immersion method	*Aspergillus niger*, *Candida albicans*, *Candida tropicalis*, *Saccharomyces cerevisiae*, *Penicillium citrinum*			Degradation of cell membrane	Zhang et al. (2008), Li et al. (2013), Oves et al. (2016), Khatoon et al. (2017)
Gold nanoparticle	Green synthesis	*Puccinia graminis tritci*, *A. niger*, *Aspergillus flavus*, *C. albicans*				Jayaseelan et al. (2013a)
Copper nanoparticle	Gel combustion method	*C. albicans*				Usman et al. (2013)
Zinc oxide nanoparticle	Green synthesis	*A. niger*, *Microsporum cannis*				El-Nahhal et al. (2020), Singh V. P. et al. (2018)
**Antiviral activity**							
Silver nanoparticle	Immersion method	HIV-1, Influenza virus, Monkey pox virus, Herpes simplex virus			Inhibition of virion binding to the cell surface	Baram-Pinto et al. (2009), Lara et al. (2010)
Gold nanoparticle	Immersion method	HIV, Influenza virus				Di Giancivincenzo et al. (2010)

### Antibacterial Activity of Different Nanoparticles

#### Silver Nanoparticles (Ag-NPs)

Different salts of silver and their derivatives are potential antimicrobial agents (Zorraquín-Peña et al., 2020). The antimicrobial properties of nanosilver particles are reported on by several researchers (Silva et al., 2017; Loo et al., 2018; Sánchez-López et al., 2020). Different mechanisms have been put forward to clarify the inhibitory impact of silver nanoparticles (Ag-NPs) on microscopic organisms (Le Ouay and Stellacci, 2015; Liao et al., 2019; Qais et al., 2019). One of the most important reasons for the antimicrobial properties of silver is high affinity towards sulphur and phosphorus. Ag-NPs react with the sulphur-containing amino acids found in the protein of bacterial cell membranes and affect the viability of bacterial cells (Roy et al., 2019). NPs react with the phosphorus moiety of the DNA and sulphur of the proteins and inhibit the DNA replication and enzymatic processes of the bacterial cell (Liao et al., 2019). Greater permeability of the cell occurs through the attachment of Ag-NPs (with a size less than 20 nm) to the sulphur-containing amino acids of the cell membrane which causes the death of the bacterial cell (Slavin et al., 2017; Guilger-Casagrande and Lima, 2019). Various studies have reported on the dose dependent-effect of Ag-NPs with the size range of 10–15 nm on the Gram-positive and Gram-negative bacteria (Pazos-Ortiz et al., 2017; Slavin et al., 2017; Chittora et al., 2020). At both high and low concentrations, silver NPs were found to inhibit the growth of bacterial cells (Wang Y. et al., 2017). In different mechanisms of inhibition of bacterial cells such as uncoupling of respiratory electron transport, blocking of respiratory chain enzymes and interference with the membrane permeability are shown by silver ions at low concentrations. Additionally, at higher concentrations, nucleic acids, and cytoplasmic contents of bacterial cells are found to be affected by silver ions (Dakal et al., 2016).

Different techniques such as TEM, SEM, and X-ray microanalyses were used to show the effect of Ag-NPs on the cell structures of Gram-positive and Gram-negative bacteria (Jung et al., 2008). Silver ion-induced almost similar morphological and physiological changes in both *E. coli*, and *Staphylococcus aureus* bacteria. But the effect of silver ion was higher in Gram-negative bacteria. It may be because of the presence of a thick layer of peptidoglycan in the Gram-positive bacteria which can prevent the inhibitory effect of silver ions up to some extent (Jung et al., 2008). The general mechanism of the death of bacterial cells by the Ag-NPs may be the interaction of silver ions to the nucleic acids. This leads to the impairment of DNA replication. Further, Smetana et al. (2008) studied the antimicrobial effect of Ag-NPs (with a size range of 2–5 nm) using green fluorescent protein (GFP)-expressing recombinant *E. coli.* Ag-NPs with a size of less than 10 nm causes perforation of the cell wall and by attaching to the bacterial cell, leads to death. Silver ions were also reported to induce reactive oxygen species in bacteria which leads to the destruction of the bacterial cell (Meena et al., 2013; Shaikh et al., 2019). It has been reported that the antimicrobial activity of Ag-NPs was enhanced by the combination of polymer even at low concentrations (Chen et al., 2016; Abbas et al., 2018; Batista et al., 2018). Chitosan, a cationic polysaccharide was used along with Ag-NPs to improve the antimicrobial properties of NPs (Abdelgawad et al., 2014). Cationic chitosan decreased the osmotic stability of the cell as well as leakage of intracellular constituents by binding with the negatively charged cell membranes.

The antimicrobial effect of chitosan Ag-NPs is much higher than its individual constituents *i.e.* chitosan and silver. Both chitosan and silver work together in chitosan Ag-NPs to destruct the bacterial cell (Regiel-Futyra et al., 2017). Chitosan attaches to the negatively charged plasma membrane of the bacteria whereas silver ions produce pores on the bacterial wall, thereby causing rapid destruction of the bacteria. Ag induced the expression of stress-related proteins such as envelope proteins and heat shock proteins on the cell membrane of the bacterial cell which has been confirmed by the proteomic approach (Zienkiewicz-Strzałka et al., 2020). Further, Tormena et al. (2020) evaluated the antimicrobial activity of Ag-NPs which were synthesized by the biological method using *Handroanthus impetiginosus* underbark extract.

#### Gold Nanoparticles (Au-NPs)

In recent decades, various investigations on the antibacterial activity of Au-NPs have been reported (Shamaila et al., 2016; Katas et al., 2019). The enhanced antimicrobial effect of Au-NPs have been demonstrated when it is used in combination with antibiotics, drugs, vaccines, and antibodies (Tao, 2018). Gu et al. (2003) reported the enhanced antibacterial effect of vancomycin antibiotic against enterococci after coating with Au-NPs. The improved efficacy of cefaclor and Au-NPs were reported against *Staphylococcus aureus* and *E. coli* when both were used together. Both cefaclor and Au-NPs show various mechanisms for inhibition of the growth of bacterial cells. Binding of Au-NPs with the DNA of the bacterial cell played an important role in the death of the bacterial cell. Cefaclor and Au-NPs both inhibited the synthesis of cell walls by creating holes which caused leakage of the contents of the bacterial cell (Rai et al., 2010). Antibacterial effects of Au-NPs were enhanced against the Gram-positive *Staphylococcus epidermidis* and the Gram-negative *Enterobacter aerogenes* when used in combination with antibiotic kanamycin. The antibacterial effect of both kanamycin and Au-NPs when used together was much higher than the individual use (Payne et al., 2016). Additionally, Rattanata et al. (2016) studied the combined effect of gallic and Au-NPs against the food borne pathogens *Plesiomonas shigelloides* and *S. flexneri* B. and demonstrated by the use of Fourier-transform infrared spectroscopy that the biomolecules of the bacterial cell were destructed by the Au-NPs–gallic acid. Further, Bagga et al. (2017) evaluated the antibacterial effect of Au-NPs along with levofloxacin antibiotic against *Staphylococcus aureus*, *E. coli* and *P. aeruginosa.* Analysis of the underlying mechanism revealed that the effect of gold nanoparticles-levofloxacin conjugate was much pronounced than when used alone (Bagga et al., 2017). A study conducted by Wongyai et al. (2020) on the antibacterial effect of greenly synthesized Au-NPs showed effective antibacterial activity against *Staphylococcus aureus*, methicillin-resistant *Staphylococcus aureus*, and *Acinetobacter baumannii*.

#### Magnesium Oxide Nanoparticles

MgO-NPs have great potential as an antimicrobial agent (Cai et al., 2018). MgO-NPs have been reported as a potential antimicrobial agent either used individually or in combination with other antimicrobial agents (Imani and Safaei, 2019). In one study, Cai et al. (2018) demonstrated the superior antibacterial properties of MgO-NPs against *R. solanacearum* at a very low concentration (250 μg/ml). Disruption of nascent biofilms and death of bacterial cell by the production of ROS, increased calcium ion concentrations and quorum sensing was reported as different antimicrobial mechanisms of the MgO-NPs against planktonic bacteria (Nguyen et al., 2018). In another study, He et al. (2016) studied the mechanism of action of MgO-NPs against some bacteria and used scanning electron microscopy technique to show the cell damage in *Campylobacter jejuni*, *E. coli*, and *Salmonella enteritidis* bacteria after treatment with MgO-NPs. The antibacterial effect of MgO-NPs was shown against *Streptococcus mutans* and *Streptococcus sobrinus* bacteria which was evident by the formation of a zone of inhibition using agar disk diffusion technique (Noori and Kareem, 2019). Similarly, Ibrahem et al. (2017) studied the role of MgO-NPs which were synthesized by the *A. niger* method as an effective antimicrobial agent against *Staphylococcus aureus* and *P. aeruginosa*. NPs synthesized by the green method proved to be effective antibacterial agents against various bacteria. MgO-NPs which were greenly synthesized by using the *Dalbergia sissoo* extract showed excellent antibacterial activity against *E. coli* and *Ralstonia solanacearum* (Khan et al., 2020).

#### Zinc Oxide Nanoparticles

ZnO-NPs are considered to be highly toxic amongst the different metallic NPs (Xie et al., 2011). Use of ZnO-NPs in agricultural and food industries is highly recommended because of selective toxicity against bacteria and negligible toxic effects on human cells (Espitia et al., 2012). Different studies have reported the antimicrobial activity of ZnO-NPs against different food-borne pathogens (Sirelkhatim et al., 2015; Khatami et al., 2018). A study conducted by Jin et al. (2009) on the antimicrobial effect of ZnO-NPs in culture media showed promising results against *Listeria monocytogenes*, *Salmonella enteritidis*, and *E. coli.* ZnO-NPs completely lysed some food borne pathogens such as *S. typhimurium* and *Staphylococcus aureus* and showed strong antimicrobial activity (Souza et al., 2019). Tiwari et al. (2018) reported the antibacterial mechanism of ZnO-NPs against *Acinetobacter baumannii* which is a multi-drug resistant pathogen. In another study, Naseer et al. (2020) used leaf extracts of *Cassia fistula* and *Melia azedarach* plants and synthesized ZnO-NPs which revealed improved antibacterial activity against *E. coli* and *Staphylococcus aureus*. Various mechanisms have been put forward to explain the antibacterial activity of the ZnO-NPs. The production of reactive oxygen species is one of the important mechanisms of the ZnO-NPs which causes lipid peroxidation and leakage of the cellular contents (Tiwari et al., 2018). ZnO-NPs also caused disruption of the cell membrane of the bacterial cell which leads to cell death (Qiu et al., 2020). Zn ions which were released from the Zn NPs were also reported to interact with the cell membrane and cellular contents of the bacterial cell (El-Nahhal et al., 2017).

#### Copper Oxide Nanoparticles (CuO-NPs)

The unusual crystal structure and high surface area make CuO-NPs an effective antimicrobial agent (Mahmoodi et al., 2018). Generally, the high concentrations of CuO-NPs are required for their better antibacterial activity (Concha-Guerrero et al., 2014). CuO-NPs were reported to possess antimicrobial activity against different bacterial pathogens such as *E. coli*, *E. faecalis*, *S. flexneri*, and *S. typhimurium* (Ahamed et al., 2014). In one study, Amiri et al. (2017) employed an agar diffusion test to assess the antibacterial properties of CuO-NPs against *Streptococcus mutans* and *Lactobacilli*. CuO-NPs exhibited effective results against both bacteria. CuO-NPs biosynthesized by using the leaf extracts of papaya, were found to have excellent antibacterial activity against a soil-borne pathogen *R. solanacearum* (Chen et al., 2019b). CuO-NPs caused damage to the cell membrane of the pathogenic bacterium and ultimately leakage of the cellular contents. It also generates toxic hydroxyl radicals which ultimately causes the death of the bacterial cell (Taran et al., 2017). CuO-NPs were biosynthesized by the actinomycetes and enhanced antimicrobial activity was reported by Nabila and Kannabiran (2018) against some bacterial pathogens. Similarly, Qamar et al. (2020) synthesized CuO-NPs from *Momordica charantia* plants with improved antibacterial activity against different bacterial pathogens such as *Bacillus cereus*, *Corynebacterium xerosis*, and *Streptococcus viridians.*

#### Aluminium Oxide Nanoparticles (Al_2_O_3_-NPs)

Aluminum oxide NPs show comprehensive applications as antimicrobial agents (Aderibigbe, 2017). Different studies have reported on the antimicrobial applications of Al_2_O_3_-NPs (Prashanth et al., 2015; Sánchez-López et al., 2020). Bala et al. (2011) prepared the alumina silver composite NPs and demonstrated the enhanced antimicrobial activity of NPs against *E. coli and Staphylococcus epidermidis.* Al_2_O_3_-NPs cause cell death by attaching to the cell surface of bacteria (Aderibigbe, 2017). In another study, Ansari et al. (2015) showed improved antibacterial activity of Al_2_O_3_-NPs against *P. aeruginosa* which were synthesized by biological methods using leaf extracts of lemongrass. Al_2_O_3_-NPs were synthesized by combustion methods and their effects were investigated against some Gram-positive and Gram-negative bacteria. The synthesized aluminium oxide NPs showed considerable effect against all the tested strains of bacteria (Prashanth et al., 2015). Further, Brintha and Ajitha (2016) prepared aluminium doped NPs and examined their antibacterial activity against some pathogenic bacteria. Similarly, Manyasree et al. (2018) studied the antibacterial activity of Al_2_O_3_-NPs against different bacteria such as *Staphylococcus aureus*, *Streptococcus mutans*, *E. coli*, and *P. vulgaris.* Green synthesized Al_2_O_3_-NPs showed enhanced antibacterial activity against Gram-positive and Gram-negative bacteria (Manikandan et al., 2019).

#### Titanium Dioxide Nanoparticles (TiO_2_-NPs)

Synthesis of metal oxide NPs *via.* chemical methods cause serious problems and are also harmful to the environment (Nayantara and Kaur, 2018). NPs synthesized by biological methods are safe, cost-effective, and environmentally friendly (Singh J. et al., 2018). Different studies have reported the synthesis of TiO_2_-NPs by biological methods called green synthesis. Green synthesis of TiO_2_-NPs has shown that the NPs synthesized by biological methods are much more effective against microbes (de Dicastillo et al., 2020). Rajakumar et al. (2012) synthesized the TiO_2_-NPs by using *A. flavus* fungus and showed enhanced antibacterial activity of TiO_2_-NPs against *E. coli*. Increased antibacterial activity of TiO_2_-NPs synthesized by using *Aeromonas hydrophila* bacterium was shown against different bacteria such as *E. coli*, *P. aeruginosa*, *Staphylococcus aureus*, *Streptococcus pyogenes*, and *E. faecalis* (Jayaseelan et al., 2013a). Subhapriya and Gomathipriya (2018) prepared the *Trigonella foenum-graecum* extract mediated TiO_2_-NPs with enhanced antibacterial activity against *Staphylococcus aureus*, *K. pneumoniae*, *E. faecalis*, *Streptococcus faecalis*, *E. coli*, *P. eruginosa*, *P. vulgaris*, *B. subtilis* and *Yersinia enterocolitica.* Bavanilatha et al. (2019) reported on the synthesis of TiO_2_-NPs by the root extracts of *Glycyrrhiza glabra* commonly known as Licorice with the help of a precursor, titanium oxysulfate. The general mechanism behind the antibacterial activity of TiO_2_-NPs is the generation of ROS. Generated ROS disrupts the cellular mechanisms of the bacteria and ultimately causes cell death. TiO_2_-NPs also interfere with the cell signaling pathways and cause changes in gene expression of the bacterial cell by affecting the transcription factors. A study conducted by Soo et al. (2020) on enhancing the antibacterial performance of TiO_2_-NPs reported the superior activity of titanium dioxide nanofibres coated with Ag-NPs as compared to intrinsic titanium dioxide nanofibres.

### Antiviral Activities of Nanoparticles

There are several studies based on the antibacterial property of metal NPs, yet the antiviral properties of metal NPs have limited reports. Some researchers have reported on the antiviral properties of different NPs (Haggag et al., 2019; Meléndez-Villanueva et al., 2019). The diseases caused by viruses present testing issues with overall social and monetary ramifications. Synthesizing antiviral drugs that can focus on the virus and maintain host cell viability is challenging (Baram-Pinto et al., 2009). Metal NPs have been proposed as antiviral systems exploiting the core material and additionally the ligands shell (Di Gianvincenzo et al., 2010). Haggag et al. (2019) studied the antiviral properties of Ag-NPs biosynthesized by *Lampranthus coccineus* and *Malephora lutea.* Green synthesized Ag-NPs showed remarkable antiviral activity against HSV-1, HAV-10, and CoxB4 virus. Khandelwal et al. (2014) reviewed the application of Ag-NPs as potential antiviral agents for different viruses. Ag-NPs have been reported to show antiviral activity against HIV-1 viruses through inhibition of CD4 dependent virion binding as well as prevention of the post-entry phase of the HIV-1 life cycle (Lara et al., 2010). Au-NPs have also been demonstrated for their role as an antiviral agent. Au-NPs biosynthesized by using garlic extracts showed potent virucidal effects against the measles virus (Meléndez-Villanueva et al., 2019).

### Antifungal Activities of Nanoparticles

Unlike the antibacterial properties of metal NPs, there are limited investigations on the antifungal activity of metal NPs. Some studies have reported on the antifungal activity of different metal NPs. Colloid Ag-NPs were reported to show antifungal activity against *A. niger* and *Penicillium citrinum* (Zhang et al., 2008). Haghighi et al. (2011) investigated the antifungal activity of TiO_2_/ZnO nanostructures against *C. albicans* and found that the TiO_2_/ZnO nanowires showed improved antifungal activity as compared to both individual NPs. A significant improvement in inhibition of growth of *A. niger* fungus was shown by the use of ZnO nanoneedles which were synthesized through the co-precipitation method (Singh J. et al., 2018). In one study, El-Nahhal et al. (2020) synthesized ZnO-NPs by the deposition onto cotton fibers and showed improved antifungal activity against *Microsporum cannis.* Further, Khatoon et al. (2017) evaluated the antifungal activity of Ag-NPs, synthesized by the tri-sodium citrate assisted chemical approach. Authors found that the Ag-NPs showed significant antifungal activity against *Saccharomyces cerevisiae* and *C. albicans* fungi. Antifungal activity of Ag-NPs prepared from the extract of a bacterial strain was demonstrated against *C. albicans* fungus (Oves et al., 2016). Jayaseelan et al. (2013b) showed improved antifungal activity of green synthesized Au-NPs by the seed extract of *Abelmoschus esculentus* plants against *Puccinia graminis*, *C. albicans*, *A. niger*, and *A. flavus.* The growth of two different species of *Candida* fungus *viz. Candida tropical* and *C. albicans* were found to be inhibited by the graphene oxide-based silver nanocomposites (Li et al., 2013). Cu-NPshave also been reported to show antifungal activity against *C. albicans* fungus (Usman et al., 2013).

### Advantages and Challenges of Nanotechnology-Based Antimicrobial Analysis

One of the promising approaches for the smart delivery of antibacterial compounds is the use of nanocarriers (Din et al., 2017). Several studies have demonstrated the advantage of antimicrobial NPs over free antimicrobial compounds (Beyth et al., 2015; Wang Y. et al., 2017; Varier et al., 2019). Stability, solubility, and side effects are the important issues of pesticide use which are reduced by nanocarriers. Nanocarriers have enabled the use of a combination of more than one antimicrobial compound into the carrier matrix (Karaman et al., 2017). The surface alterations can be completed by focusing on ligands on the nanocarriers that are not known by the immune system and instead are explicitly focused on unique microbes. The organization of antimicrobial agents utilizing NPs can enhance the general pharmacokinetics by advancing the therapeutic index, broadening drug circulation, and maintaining controlled drug discharge. Many pathogenic bacteria develop antibacterial resistance which is prevented by the use of antibacterial NPs (Baptista et al., 2018). Bacteria finds it very difficult to develop resistance against antibacterial NPs because of the modularity in their design. Antibacterial NPs are composed of an antibacterial core material (e.g. metal or metal oxide) surrounded with an antibacterial polymeric shell or coating, in which antibiotic drugs could be loaded (Lam et al., 2016). Wu et al. (2016) reported the destruction of bacterial cell walls through a nano-piercing process after the dissolution of the polymeric shells by the core material of zinc dopped copper oxide antibacterial NPs. Differing opportunities for combination therapy along with existing antimicrobials to arrive at synergistic impacts are clear. In spite of the fact that NP-based antibacterial treatments guarantee huge advantages and advances in tending to the key obstacles in treating infectious diseases, there are difficulties in interpreting this energizing innovation for clinical use (Karthikeyan et al., 2016). These incorporate assessing the collaborations of NPs with cells, tissues, and organs, which as needs be recalibrates dosages and distinguishes legitimate organization courses to acquire therapeutic impacts. Henceforth, to give a clinical interpretation of nanomaterials, normalized *in vitro* experimentations that will give *in vivo* applicable information ought to be built up (Huh and Kwon, 2011).

### Biostimulants

Biostimulants are natural or artificial substances, generally used for the improvement of the quality of the plants. They promote plant growth, increase tolerance against biotic and abiotic stresses, and enhance the yield and quality of crop plants. The need for fertilizers has also reduced because of use of biostimulants (Rouphael and Colla, 2020). NPs can also be used as biostimulants as they enhance the quality of crops. Several studies have reported on the biostimulant properties of different NPs (Byczyńska, 2017; Juárez-Maldonado et al., 2019; Kumaraswamy et al., 2019). Van et al. (2013) demonstrated the increase in the chlorophyll content, net photosynthetic rate, and nutrient uptake in coffee plants after treatment with CS-NPs. Further, Kumaraswamy et al. (2019) reported the biostimulant properties of the salicylic acid chitosan nanoparticles (SA-CS NPs) for promoting plant growth and defense mechanisms in maize. Different mechanisms such as elevation of antioxidant-defense enzyme activities, balancing of reactive oxygen species (ROS), and cell wall reinforcement by lignin deposition were used by SA-CS NPs to enhance the growth and defense system of the maize plants. Selenium nanoparticles (Se-NPs) biosynthesized by *Trichoderma* spp. showed growth-promoting characters in *Vigna radiata* plants (Keswani et al., 2014; Keswani et al., 2016; Bărbieru et al., 2019). Venkatachalam et al. (2017) studied the plant growth-promoting role of phycomolecules coated ZnO-NPs with phosphorus (P) supplementation in cotton and observed that the combination of bioengineered ZnO-NPs with P supplementation resulted into an increase in biomass, photosynthetic pigments, total soluble proteins, and antioxidant enzyme activities. Nano-silver also possesses the plant growth-promoting characteristics which can be used as a potential plant biostimulant (Byczyńska, 2017).

### Pesticide Carriers

It has been estimated that almost 90% of applied pesticides are lost due to leaching, evaporation, and degradation (Lushchak et al., 2018). The loss of pesticides causes environmental pollution and increases the cost of pest management. The use of NPs as pesticide carriers have many advantages *viz.*, enhanced bioavailability, improved specificity, ease and safety in handling, minimum ecological damage, and lower application rates (Worrall et al., 2018). Different NPs as pesticide carriers are listed in Table 2. The increased cost and toxicity of low water-soluble insecticides can be minimised by the use of NPs as carriers which can increase the solubility (Campos et al., 2018). Several studies have reported the use of NPs for the smart delivery of various insecticides (Lu et al., 2013; Zhang et al., 2013; Wang Y. et al., 2014; Campos et al., 2018). Lu et al. (2013) demonstrated the role of CS-NPs as a carrier for azadirachtin for the sustained release of insecticide. An increase in uptake and higher toxicity of thiamethoxam insecticide was reported against *Helicoverpa armigera* larvae when intercalated with dendrimer NPs (Liu X. et al., 2015). In another study, Nguyen et al. (2014) showed an increase in toxicity of layered double hydroxides (LDH) NPs encapsulated with anacardic acid against *Spodoptera litura.* Anacardic acid alone did not show higher mortality against *S. litura* but after loading with LDH NPs, an improvement in toxicity was observed. Evaporation of the active molecules of the pesticides after an application is a common problem associated with the loss of pesticides. Essential oils show insecticidal properties but such properties rapidly evaporate due to their chemical instability in the presence of air, light, moisture, and high temperatures. A decrease in evaporation of *Artemisia arborescens* L. essential oil was reported when encapsulated with solid lipid NPs (Lai et al., 2006). Further, Yang et al. (2009) reported the increase in mortality rate from 11 to 80% of essential oil of garlic intercalated with polyethylene glycol NPs against red flour beetles (*Tribolium castaneum*) in rice plants. In another study, α- pinene and linalool were loaded into silica NPs and applied to castor leaves and then infested with *S. litura* and castor semi looper. It was found that both insects showed lower feeding activity on treated castor leaves and ultimately led to death due to starvation (Rani et al., 2014). The stability of the active molecules of the insecticides is also an important concern because it decreases the use of insecticides which is essential for environmental health.

**TABLE 2:** Different nanoparticles as carriers of various pesticides (fungicides, insecticides and herbicides).

Carriers of fungicides					
Nanoparticles	Methods of synthesis		Fungicides	Target organism	References
Polymeric nanoparticles (Polyvinylpyridine and polyvinylpyridine-co-styrene as a polymer)	Interfacial polymerization		Tebuconazole and Chlorothalonil	*Gloeophyllum trabeum*	Liu et al. (2001, 2002)
Polymeric nanoparticles (Polyvinylpyridine and polyvinylpyridine-co-styrene as a polymer)	Interfacial polymerization		Tebuconazole, Chlorothalonil, and KATHON 930	*Trametes versicolor*, *Gloeophyllum trabeum*	Liu et al. (2002)
Bacterial ghost from Pectobacterium cypripedii	Bacterial ghost technology		Tebuconazole	*Erysiphe graminis*, *Leptosphaeria nodorum*, *Pyrenophora teres*, *Sphaerotheca fuliginea*	Hatfaludi et al. (2004)
Porous hollow silica nanoparticles	Surfactant templating method		Validamycin	*–*	Liu et al. (2006)
Nano sized calcium carbonate	Reversed-phase microemulsion method		Validamycin	*Rhizoctonia solani*	Qian et al. (2011)
Porous hollow silica nanospheres	Miniemulsion method		Tebuconazole	*–*	Qian et al. (2013)
Polylactic acid nanoparticles	Electrospinning method		Crude extraxt of *Chaetomium globosum* and *Chaetomium cupreum*	*–*	Dar and Soytong (2014)
Mesoporous silica nanospheres	Sol-gel process		Metalaxyl		Wanyika, (2013)
Chitosan-Lactide Copolymer Nanoparticles	Nano-precipitation method		Pyraclostrobin	*Colletotrichum gossypii* Southw	Xu et al. (2014)
Chitosan-polylactide (CS-PLA) graft copolymer nanoparticles	Nano-precipitation method		Flusilazole	*–*	Mei et al. (2014)
Solid lipid nanoparticles	Solvent evaporation method		Carbendazim and Tebuconazole	*–*	Campos et al. (2015)
Mesoporous silica nanoparticles	Encapsulation method		Allyl isothiocyanate, Carvacrol, Cinnamaldehyde, Diallyl disulfide, Eugenol, Thymol, and Thymoquinone	*Aspergillus niger*	Janatova et al. (2015)
Lecithin/Chitosan nanoparticles	Ionic interaction method		Kaempferol	*Fusarium oxysporum*	Ilk et al. (2017)
Solid lipid nanopartilces	High shear homogenization and Ultra sound technique		Zataria multiflora essential oil	*Aspergillus ochraceus*, *A. niger*, *A. flavus*, *Alternaria solani*, *Rhizoctonia solani*, and *Rhizopus stolonifer*	Nasseri et al. (2016)
Engineered gold nanoparticles	Encapsulation method		Ferbam	*–*	Hou et al. (2016)
Chitosan capped mesoporous silica nanoparticles	Liquid crystal templating method		Pyraclostrobin	*Phomopsis asparagi*	Cao et al. (2016)
Polymeric nanoparticles	Ionic interaction method		Carbendazim	*Fusarium* oxysporum, *Aspergillus parasiticus*	Kumar et al. (2017)
Mesoporous silica nanoparticles	Sol-gel process		Pyrimethanil	*–*	Zhao X. et al. (2017)
Mesoporous silica nanoparticles	Sol-gel process		Prochloraz	*Botrytis cinerea*	Zhao et al. (2018)
Chitosan nanoparticles	Emulsion-ionic gelation method		Clove essential oil	*Aspergillus niger*	Hasheminejad et al. (2019)
Silver nanoparticles	Encapsulation method		*Ginkgo* fruit extract	*Bipolaria maydis*	Huang et al. (2018)
Chitosan nanoparticles	Emulsion-ionic gelation method		*Cymbopogon martinii* essential oil	*Fusarium graminearum*	Kalagatur et al. (2018)
Polybutylene succinate and polylactic acid nanoparticles	Solvent evaporation method		Azoxystrobin, Difenoconazole	*–*	Wang X. et al. (2018)
Mesoporous silicananoparticles	Selective etching strategy and subsequent annealing treatment		Pyraclostrobin	*Phomopsis asparagi*	Cao et al. (2018)
**Carrier of insecticides**					
**Nanoparticles**	**Methods of synthesis**		**Insecticides**	**Target organism**	**References**
Solid lipid naoparticles	High pressure homogenization technique		*Artemisia arborescens* L. essential oil	*Bemisia tabaci*	Lai et al. (2006)
Porous hollow silica nanoparticles (PHSNPs)	Sol-gel method		Avermectin	*–*	Wen et al. (2005), Li et al. (2006), Li et al. (2007)
Polyethylene glycol (PEG) coated nanoparticles	Melt-dispersion method		Garlic essential oil	*Tribolium castaneum*	Yang et al. (2009)
Chitosan-coated beeswax solid lipid nanoparticles (CH-BSLNPs)	Hot homogenization and Sonication method		Deltamethrin	*–*	Nguyen et al. (2012a)
Nanostructured lipid carriers (NLCs)	Hot homogenizationand Sonication method		Deltamethrin	*–*	Nguyen et al. (2012b)
Silica nanoparticles	Sol-gel process		Chlorfenapyr	Cotton Bollworm larva	Song et al. (2012)
Carboxymethyl chitosan with ricinoleic acid (R-CM-chitosan) nanoparticles	Emulsion ionic gelation method		Azadirachtin	–	Feng and Peng (2012)
Chitosan copolymer nanoparticles	Solvent evaporation method		Chlorpyrifos	–	Zhang et al. (2013)
Octahydrogenated retinoic acid-conjugated glycol chitosan nanoparticles	Chemical Conjugation method		Azadirachtin	Tobacco cutworm culture	Lu et al. (2013)
Sodium alginate nanoparticles	Emulsion cross linking technology		Imidacloprid	Leafhoppers	Kumar et al. (2014)
Silica nanoparticles	Immersion method		α-Pinene and Linalool	*Spodoptera litura* F., *Achaea janata* L.	Rani et al. (2014)
Porous silica nanoparticles	Hydrophilic delivery method		Abamectin	*–*	Wang Y. et al. (2014)
MgAl layered double hydroxide nanoparticles	Solvent evaporation method		Anacardic acid	*Spodoptera litura*	Nguyen et al. (2014)
Silica nanocapsules	Bio-inspired templating platform technology		Fipronil	Termites	Wibowo et al. (2014)
Polydopamine microcapsule	Emulsion interfacial-polymerization method		Avermectin	–	Jia et al. (2014) Sheng et al. (2015)
Dendrimer-based nanocarrier	Conjugation method		Thiamethoxam	*Heliothis armigera*	Liu et al. (2012)
Nano sized capsule	Encapsulation method		Pyrethroid	*Danio rerio*	Meredith et al. (2016)
Polymer-coated silver nanoparticles	Immersion method		Organochlorine	*–*	Glinski et al. (2016)
Silver nanoparticles	Conjugation method		*Suaeda maritima* leaf extract	*Aedes aegypti*, *Spodoptera litura*	Suresh et al. (2018)
Chitosan nanoparticles	Cross-linking technology		Ponneem	*Heliothis armigera*	Paulraj et al. (2017)
Bioinspired nanoparticles	Solvent evaporation technology		Avermectin		Liang et al. (2017)
Castor oil-based polyurethanes	Emulsion solvent evaporation method		Avermectin		Zhang et al. (2017)
Chitosan and Zinc oxide based nanoparticles	Sol-gel and Ion tropic gelation technique		Azadirachtin	*Caryedon serratus* O.	Jenne et al. (2018)
β-cyclodextrin nanoparticles	Kneading method		Carvacrol and Linalool	*Tetranychus urticae, Helicoverpa armigera*	Campos et al. (2018)
Chitosan/gum arabic nanoparticles	Encapsulation method		Geraniol	*Bemisia tabaci*	de Oliveira et al. (2018a)
Chitosan/tripolyphosphate nanoparticles	Encapsulation method		*Satureja hortensis* L.	*Tetranychus urticae* Koch	Ahmadi et al. (2018)
Zein nanoparticles	Anti-solvent precipitation method		Geraniol and *R*-citronellal	*Tetranychus urticae* Koch	de Oliveira et al. (2018b)
Chitosan/sodim tripolyphosphate nanoparticles	Encapsulation method		Nicotine hydrochloride	*Musca domestica*	Yang et al. (2018)
Hybrid magnetic nanocomposites	Chemical bonding approach		Benzenoid	*–*	Wang Y. et al. (2018)
α-Amylase and α-cyclodextrin based hollow mesoporous silica nanoparticles	Encapsulation method		Avermectin	*Plutella xylostella*	Kaziem et al. (2018)
**Carrier of herbicides**					
**Nanoparticle**	**Methods of synthesis**	**Herbicide**		**Target organism**	**References**
Polymer montmorillonite nanoparticles	Solution and solid state reaction methods	Paraquat (PQ; 1,1′-dimethyl-(4,4′-bipyridium) dichloride)		**–**	Han et al. (2010)
Alginate/Chitosan nanoparticles	Solution and solid state reaction methods	Paraquat		**–**	dos Santos Silva et al. (2011)
Manganese carbonate core shell nanoparticles	Hydrothermal/solvolthermal method	Pendimethalin		**–**	Kanimozhi and Chinnamuthu (2012)
Polymeric poly (ɛ-caprolactone) nanocapsules	Interfacial polymerization method	Ametryn, Atrazine, and Simazine		**–**	Grillo et al. (2012)
Chitosan/tripolyphosphate nanoparticles	Ionic gelification technique	Paraquat		**–**	Grillo et al. (2014, 2015)
Solid lipid nanoparticles	Emulisfication and solvent evaporation method	Simazine and Atrazine		*Raphanus raphanistrum*	de Oliveira et al. (2015)
Nanosized tubular halloysite and platy kaolinite	Encapsulation method	Amitrole		–	Tan et al. (2015)
Alginate/chitosan and chitosan/tripolyphosphate nanoparticles	Ionotropic gelification method	Imazapic and Imazapyr		*Bidens pilosa*	Maruyama et al. (2016)

Several studies have reported the incorporation of different fungicides with NPs and their enhanced activity against different fungi. Various problems associated with fungicides such as low-water-solubility, volatilization, and stability were resolved by the loading of fungicides into NPs. Hatfaludi et al. (2004) reported increased toxicity by the use of fluorescence-labeled *Pectobacterium cypripedii* ghosts as a carrier of fungicide tebuconazole against different fungal pathogens such as *Erysiphe graminis*, *Leptosphaeria nodorum*, *Pyrenophora teres*, and *Sphaerotheca fuliginea.* An increase in inhibition against *Colletotrichum gossypii* Southw was also seen when pyraclostrobin was intercalated into chitosan-lactide copolymer NPs (Xu et al., 2014). Pyraclostrobin loaded NPs showed improved fungicidal activity against *Colletotrichum gossypii* Southw after long post-treatment which further presented controlled release properties. In another study, Ilk et al. (2017) used lecithin/CS-NPs to improve the inhibition efficacy of kaempferol fungicide against *Fusarium oxysporum*. Several studies have reported the improvement of the low solubility of tebuconazole and chlorothalonil fungicides by loading into various kinds of NPs. Liu et al. (2001) successfully incorporated tebuconazole and chlorothalonil into polymeric NPs and treated southern pine sapwood samples. It was found that the treated samples indicated enhanced resistance against the wood decay fungus *Gloeophyllum trabeum.* Further, Liu et al. (2002) created the smaller and more stable surfactant free NPs after loading with chlorothalonil and tebuconazole which ultimately increased the uptake into the wood. It also showed high inhibition activity against *G. trabeum* and *Trametes versicolor*.

Volatilization of essential oils with fungicidal activity is an important issue regarding the use of fungicides. Some essential oil components with antifungal activity, such as allyl isothiocyanate, carvacrol, cinnamaldehyde, diallyl disulfide, eugenol, thymol, and thymoquinone were encapsulated into mesoporous silica NPs. Encapsulated compounds showed enhanced activity against *A. niger* and also showed long-term effects by controlled release and ease of application as compared to fungicides alone (Janatova et al., 2015). Further, Nasseri et al. (2016) loaded *Zataria multiflora* essential oil into solid lipid NPs and showed enhanced efficacy against different fungal pathogens such as *Aspergillus ochraceus*, *A. niger*, *A. flavus*, *Alternaria solani*, *R. solani*, and *Rhizopus stolonifer.* Another major pesticide issue is the movement of water and chemicals through soil called leaching which affects the usage of pesticides. Metalaxyl fungicide encapsulated with MSN was investigated for the reduced loss and changed release profile. MSN entrapped fungicides showed controlled release behaviour as about only 11.5% of the free metalaxyl was released into the soil over a time period of 30 days as compared to the free fungicides in which 76% was released within the same time period (Wanyika, 2013). Campos et al. (2015) prepared two different types of NPs, polymeric, and solid lipid NPs loaded with a combination of tebuconazole and carbendazim and investigated their controlled release behavior and storage properties. After the loading of fungicides with NPs, their release profile and toxicity was changed. Slow controlled release, enhanced stability and fungicidal activity against *R. solani* were seen in validamycin loaded nanosized calcium carbonate as compared to fungicide alone (Qian et al., 2011). Further, Kumar et al. (2017) studied the bio-efficacy of polymeric NPs loaded with carbendazim against *F. oxysporum* and *Aspergillus parasiticus* and found that after incorporation of fungicide with NPs, the inhibition activity was enhanced. Moreover, Zhao X. et al. (2017) studied the uptake and distribution of the pyrimethanil loaded mesoporous silica NPs in cucumber plants and reported lower accumulation of fungicide loaded NPs in the edible parts of the plants.

Herbicides play a vital role in integrated weed management programs. The major concern of the herbicides is their non-target toxicity. Encapsulation of herbicides with NPs provides a better solution for the non-target toxicity of the herbicides. The development of NP-based herbicides has also included a wider variety of NPs. Maruyama et al. (2016) reported the improved mode of action and reduced toxicity of Imazapic and Imazapyr herbicides after loading with CS-NPs. Authors also studied the effect of herbicide loaded NPs on the soil microbiota and found no changes in the number of soil bacteria. An increase in physicochemical stability and high encapsulation efficiencies were reported in solid lipid NPs loaded with simazine and atrazine herbicides (de Oliveira et al., 2015). Herbicide loaded NPs showed enhanced toxicity and no activity against target *Raphanus raphanistrum* and non-target plants (*Zea mays*), respectively, as compared to herbicides alone. Further, de Oliveira et al. (2016) compared the effects of the clomazone herbicide in both its free form and associated with chitosan-alginate NPs. Loading of herbicides with NPs also reduces the problem of leaching.

Additionally, Chidambaram (2016) used rice husk nanosorbents encapsulated with 2, 4-dichlorophenoxyacetic acid herbicide and showed the controlled release profile, reduced leaching activity and enhanced toxicity against the target plant (*Brassica* sps.) as compared to herbicides alone. The release profile, stability, and storage were seen to be improved in the alginate/CS-NPs intercalated with paraquat herbicide (dos Santos Silva et al., 2011). The behaviour of herbicides in terms of chemical stability, solubility, bioavailability, photodecomposition, and soil sorption was changed by the incorporation of herbicides into the poly (ɛ-caprolactone) nanocapsules (Grillo et al., 2012). Further, Grillo et al. (2014) prepared herbicide atrazine loaded with poly (ɛ-caprolactone) nanocapsules and showed the increased physico-chemical stability and herbicide release profile. Herbicide paraquat was also encapsulated into CS-NPs by Grillo et al. (2015) and they reported the increased stability and reduced non-target toxicity of the herbicides.

### Internet of Nano Things (IoNT)

It has been discussed in the above sections that nanotechnology is considered as an upfront technology to design and develop nanometers scale devices. The Internet of Things (IoT) is the intelligent interaction of different sensors and the main application of IoT has been discussed in the field of nanotechnology to offers effective solutions and opportunities in the area of pharmaceutical industries, agriculture, military and computing systems (Atlam et al., 2018). The IoNT (internet of nano things) is the replacement of sensors by nanosensors, which established a new aspect of IoT in the field of nanotechnology. Therefore, the interconnection of nanodevices and nanosensors with the internet contains a light-emitting diode referred to as IoNT (Internet of Nano Things). IoNT is introduced by Akyildiz and Jornet (Akyildiz and Jornet, 2010) where it is operated by terahertz frequencies using graphene-based nano-antennas. The dimension of this nanomachine ranges between 1 and 100 nm (Chaudhry et al., 2017; Miraz et al., 2018). According to the (UN DESA report, 2015) (UN DESA report ‘World Population Prospects: the 2015 revision), the world population is estimated to reach 9.7 billion by 2050 which will cause severe food scarcity. Fortunately, IoNT will open-up the domain in the agricultural field with more confidence to produce more adequate food supplies by enhancing crop production. IoNT reduces the harmful influences on the environment significantly (Patil et al., 2012; Nida et al., 2015; Maksimović and Omanović-Mikličanin, 2017). The use of IoNT enhances the utilization of inputs in agriculture such as water, soil, pesticides, fertilizers, etc., reduces production costs, creates high profitability, and ensures sustainability and environmental protection. Even with more future adaptability aspects, the IoNT faces several challenges due to privacy and security concerns. IoNT uses two systems of communication: Electromagnetic Nano-Communication and Molecular Communication (Lakshmi, 2018). Therefore, through the development of nanomachines, IoNT has a wonderful impact and significantly adds to revolutionizing agriculture practices to make the food industry more efficient, sustainable, and safe (Maksimović and Omanović-Mikličanin, 2017). There is a wide range of IoNT applications that have been reported. IoNT can be applied in a body sensor network (BSN) in which it plays a crucial role in the collection of data on the biological activity of patients. It also can be applied for environmental monitoring, such as temperature or air pollution. (Nayyar et al., 2017). In agriculture, it develops various exactitude agricultural practices which leads to the growth of crops and monitoring of animal, grass or pesticide and insecticide (Balasubramaniam and Kangasharju, 2013; Jarmakiewicz and Parobczak, 2016; Nayyar and Puri, 2016; Nayyar et al., 2017).

### Future Perspectives

In this review, we have discussed detailed information about NPs such as their definition, types, synthesis, characterizations, properties, and applications. Several studies have shown that nanotechnology plays a very important role in commercial development. It is improving the everyday lives of human beings by increasing their performance and competence with daily objects. This technology has been used to provide a safe environment by improving air and water quality and also provides renewable energy sources for a sustainable future. We need to find more breakthroughs and novel prospects for advances in nanotechnology to develop the world economy. NPs are used in several fields such as agriculture, electronics, food, medical diagnostics, and pharmaceutical industries. This review discussed the roles of nanomaterials to show their great promise in agricultural fields. The interaction of plants with NPs results in various changes in morphological parameters, physiological parameters, and at the genotoxic level. It helps in the growth of plants through changes in their metabolic processes. It is being focused on to enhance the targeted delivery of fertilizers and pesticides and is used to minimize waste production through nano-based approaches. Currently, engineered NPs have been used widely to enhance crop production. Through nanotechnology, crop disease suppression can be explored adequately to enhance crop production. As we have discussed, apart from crop production NPs can also be used for medical diagnosis due to their surface chemistry, biocompatibility, stability, and regulating toxicity in biological systems. Therefore, nanotechnology needs to be studied intensively to analyse its long-term toxicity. The overall studies have stated that the application of NPs need more optimization for synthesis, mechanisms, and biofunctionalization of NPs. These NPs as nanobiosensors may improve plant development by detecting phytoregulators and secondary metabolites. Even with more studies, genome editing still remains an immense challenge, therefore with the help of NPs, CRISPR-Cas9 technology will provide great innovations to plant genetics. Significant research should be dedicated to this field; it will result in great benefit to plants as well as humans, and create more efficient and environmentally friendly approaches. Apart from that, most of endophytic microorganisms are unexplored and uncultivated, therefore it’s very important to focus research on developing innovative processes for the identification and isolation of the endophytic microorganisms in the green synthesis of metal NPs.

## Glossary

Ag: Silver
Ag_2_O: Silver oxide
Ag-NPs: Silver nanoparticles
Al: Aluminium
Al_2_O_3_: Aluminium oxide
Al_2_O_3_-NPs: Aluminium oxide nanoparticles
Au: Gold
Au-NPs: Gold nanoparticles
Bi: Bismuth
Bi_2_O_3_: Bismuth sesquioxide
CaO: Calcium oxide
Cd: Cadmium
CLS: Curvularia leaf spot
CNT: Carbon nanotubes
Co: Cobalt
CS-NPs: Chitosan nanoparticles
CTAB: Cetyl trimethylammonium bromide
Cu: Copper
Cu-NPs: Copper nanoparticles
CuO: Copper oxide
CuO-NPs: Copper oxide nanoparticles
CVD: Chemical vapour deposition
DTT: Dithiothreitol
Fe: Iron
FeO: Ferrous oxide
GFP: Green fluorescent protein
GNPs: β-d-Glucan nanoparticles
IgA: Immunoglobulin A
IoNT: Internet of nano things
ISR: Induced systemic resistance
LDL: Low density lipoprotein
Mg: Magnesium
MnO_2_: Manganese dioxide
MgO: Magnesium oxide
MgO-NPs: Magnesium oxide nanoparticles
MLVs: Mutilamellar vesicles
MSCs: Mesenchymal stem cells
MSN: Mesoporous silica nanoparticle
Ni: Nickel
NNI: National nanotechnology initiative
NPs: Nanoparticles
PAL: Phenylalanine ammonia lyase
Pb: Lead
Pd: Palladiumn
POX: Peroxidases
PRSV: papaya ring spot virus
Pt: Platinum
ROS: Reactive oxygen species
Sb: Antimony
SDR: Spinning disc reactor
SDS: Sodium dodecyl sulphate
Ta: Tantalum
Ti: Titanium
TiO_2_: Titanium dioxide
TiO_2_-NPs: Titanium dioxide nanoparticles
UVs: Unilamellar vesicles
Zn: Zinc
ZnO: Zinc oxide
ZnO-NPs: Zinc oxide nanoparticles
